# Reconstructing firm-level interactions in the Dutch input–output network from production constraints

**DOI:** 10.1038/s41598-022-13996-3

**Published:** 2022-07-13

**Authors:** Leonardo Niccolò Ialongo, Camille de Valk, Emiliano Marchese, Fabian Jansen, Hicham Zmarrou, Tiziano Squartini, Diego Garlaschelli

**Affiliations:** 1 IMT School for Advanced Studies, Lucca, 55100 Italy; 2 Scuola Normale Superiore, Pisa, 56126 Italy; 3 ABN AMRO Bank N.V., Amsterdam, 1082 PP The Netherlands; 4 Lorentz Institute for Theoretical Physics (LION), University of Leiden, Leiden, 2333 CA The Netherlands; 5 ING Bank N.V., Amsterdam, 1102 CT The Netherlands; 6 Institute for Advanced Study (IAS), University of Amsterdam, Amsterdam, 1012 GC The Netherlands

**Keywords:** Complex networks, Information theory and computation

## Abstract

Recent crises have shown that the knowledge of the structure of input–output networks, at the firm level, is crucial when studying economic resilience from the microscopic point of view of firms that try to rewire their connections under supply and demand constraints. Unfortunately, empirical inter-firm network data are protected by confidentiality, hence rarely accessible. The available methods for network reconstruction from partial information treat all pairs of nodes as potentially interacting, thereby overestimating the rewiring capabilities of the system and the implied resilience. Here, we use two big data sets of transactions in the Netherlands to represent a large portion of the Dutch inter-firm network and document its properties. We, then, introduce a generalized maximum-entropy reconstruction method that preserves the production function of each firm in the data, i.e. the input and output flows of each node for each product type. We confirm that the new method becomes increasingly more reliable in reconstructing the empirical network as a finer product resolution is considered and can, therefore, be used as a realistic generative model of inter-firm networks with fine production constraints. Moreover, the likelihood of the model directly enumerates the number of alternative network configurations that leave each firm in its current production state, thereby estimating the reduction in the rewiring capability of the system implied by the observed input–output constraints.

## Introduction

The structure of the input–output network formed by the superposition of multiple supply chains has important implications for the economy^1^. Extreme weather events that disrupted supply chains in a given region have been shown to propagate economic distress over this network, indirectly affecting the whole economy^2,3^. The constraints that the input–output specificity of firms imposes on the economy can transform micro-economic shocks into fluctuations of aggregate demand and supply^4^. This has been further highlighted by the recent COVID-19 pandemic, where the structure of the production network has driven the propagation of negative economic shocks as a consequence of the lack of demand or of the limitations in the supply of intermediate products^5^.

There are several models that attempt to understand how simple firm-level interactions can lead to a complex behaviour of the aggregate: from agent-based models^6^ to simple inventory dynamics^7^. In most cases, however, these studies are either conducted at the aggregate industry level or with unrealistic, simulated network structures. This is mostly due to the lack of access to reliable data on firm-level interactions: in fact, input–output relationships, at the level of firms, are difficult to observe due to the sensitive nature of the information and, for most countries, to the absence of a dataset capturing the structure of these same relationships in a reliable way. To the best of our knowledge, few notable exceptions exist, some of which have been studied more extensively than others: in the US, the SEC requires companies to list their major customers^8^; in Belgium^9^ and Hungary^10^ VAT tax declarations have been used to construct production networks; in Japan, there are two large commercial datasets, from Tokyo Shoko Research Ltd. and from Teikoku Databank Ltd., that have been used in numerous studies^2,11,12^.

Given the limited availability of firm-level data, it is important to develop a statistical reconstruction framework capable of reproducing the structural properties of real inter-firm networks from partial information. Different approaches to the topic of supply chains reconstruction have appeared in the literature. A first one tries to reconstruct inter-firm networks by matching known inter-sector flows in a deterministic fashion^13^; a second one uses machine-learning algorithms to predict edges based on the firms features^14–16^; a third one prescribes to employ available, external data to infer supply relationships between firms—in the case-study of Hungary, mobile phone data have been used^17^. The approach we adopt here rests upon the rich toolbox of statistical physics.

Maximum-entropy graph ensembles have been successfully employed to reconstruct features of real-world economic networks^18^. As it has been shown by several independent ‘horse-races’^19–23^, this analytical approach outperforms competing probabilistic recipes in providing an accurate prediction of a set of network features, ranging from the microscopic (e.g. true positives, true negatives) to the macroscopic ones (e.g. the trend of the average nearest neighbours degree, or that of the clustering coefficient, as a function of the nodes degree)^18^. In the case of inter-bank networks, a very efficient algorithm has been shown to perform similarly to the Configuration Model, even in absence of degree information. This model, named density-corrected Gravity Model (dcGM)^24^, entails a two step recipe that first draws links and then assigns them weights using only the strengths of each node and the total number of links of the graph^25^. The dcGM with exponentially distributed weights ($\text {CReM}_B$ in the paper, hereby simply dcGM) has been shown to provide optimal performance while keeping the computational complexity low^26^.

This model, although preserving the nodes out- and in-strengths and the density of the graph, does not constrain any network mesoscopic feature which may be known to play an important role—e.g. the presence of sectoral interdependencies, as in the case of inter-firm networks. In fact, the dcGM has been developed in a context where the nature of the exchange is the same for every actor, hence where a link always represents a credit relationship. When firm-to-firm relationships are considered, instead, the nature of these connections is necessarily heterogeneous as the kind of goods and services exchanged will vary significantly between agents: naturally, this cannot be replicated by a model that does not constrain any property of the kind. Our contribution is devoted to extending the dcGM to incorporate the available information on sector linkages: from an economic point of view, these quantities reflect constraints ‘affecting’ the production of firms. To the best of our knowledge, this is the first maximum-entropy reconstruction model that explicitly embodies this kind of production constraints.

## Methodology

Let us represent a directed, weighted network via an asymmetric adjacency matrix *W* whose generic entry $w_{ij}$ is the weight of the connection from node *i* to node *j*. In turn, *W* induces the binary adjacency matrix *A* where $a_{ij}=1$ if a connection from node *i* to node *j* is present and $a_{ij}=0$ otherwise. In what follows, we use the $*$ symbol to mark the original graph, as in $A^*$ and $W^*$, and the empirical values of the objective quantities. An important observation concerns self-loops that, as usual, are not allowed: their removal introduces a small distortion on the expected value of the strengths (see^26^ for a detailed discussion) that, however, does not affect the overall performance of the method.

### The density-corrected gravity model (dcGM)

The density-corrected Gravity Model belongs to the family of Exponential Random Graphs (ERGs). The latter are probabilistic graph ensembles that are derived from the maximum-entropy principle^27^, a well established method for inference given a set of constraints on the ensemble properties. The application of this principle to networks^28^ leads to different models, each one induced by the constrained set of properties: the parametric models thus obtained can be then fitted to real data invoking the maximum likelihood principle^29^.

The dcGM is derived from the Directed Configuration Model (DCM), an ERG model where the out- and in-degree sequences play the role of constraints. The dcGM is obtained by replacing the information provided by degrees, often unavailable in practice, with a ‘fitness ansatz’ that rests upon the assumption that the degrees themselves are proportional to strengths (for a detailed discussion about the relationship between ERGs and the dcGM see section S.2 in the Supplementary Information).

The density-corrected Gravity Model is a two-step probabilistic recipe. At the first step, each edge $a_{ij}$ is sampled independently, according to a Bernoulli distribution with parameter $p_{ij}^\text {dcGM}$ that represents the probability for any two nodes *i* and *j* to connect

1 $$\begin{aligned} p_{ij}^\text {dcGM}=\frac{zs_i^\text {out}s_j^\text {in}}{1+zs_i^\text {out}s_j^\text {in}},\quad \forall \,i\ne j \end{aligned}$$

where $s_i^\text {out} = \sum _{j(\ne i)} w_{ij}$ is the out-strength of node *i* and $s_j^\text {in} = \sum _{i(\ne j)} w_{ij}$ is the in-strength of node *j*. The only parameter of the model, *z*, can be determined by solving the equation

2 $$\begin{aligned} \langle L \rangle = \sum _{i}\sum _{j(\ne i)}p_{ij}^\text {dcGM}=\sum _{i}\sum _{j(\ne i)}a^*_{ij}=L^* \end{aligned}$$

that fixes the expected link-density of the network.

The second step of the model embodies a prescription to determine weights. In its simplest version, it deterministically assigns each sampled edge (i.e. every *i*, *j* pair such that $a_{ij}=1$) the weight $w_{ij}=s_i^\text {out}s_j^\text {in}/(w^\text {tot}\cdot p_{ij}^\text {dcGM})$ where $w^\text {tot}=\sum _is_i^\text {out}=\sum _is_i^\text {in}$ is the total weight of the network. Such a prescription can be further refined by letting the weights of the sampled edges be drawn from either a geometric distribution $w_{ij}\sim \text {Geo}(\beta _{ij})$ or an exponential one $w_{ij}\sim \text {Exp}(\beta _{ij})$, according to the nature of weights (discrete, in the first case or continuous, in the second one). Notice that while the Bernoulli prescription ensures that

3 $$\begin{aligned} \langle w_{ij}\rangle =\frac{s_i^\text {out}s_j^\text {in}}{w^\text {tot}},\quad \forall \,i\ne j \end{aligned}$$

hence allowing us to recover the expected weight defining the Gravity Model, in the other two cases the parameters of the distribution must be chosen to ensure that the condition above holds: specifically, $\beta _{ij}=(s_i^\text {out}s_j^\text {in}-w^\text {tot}\cdot p_{ij}^\text {dcGM})/s_i^\text {out}s_j^\text {in}$ for the geometric distribution and $\beta _{ij}=w^\text {tot}\cdot p_{ij}^\text {dcGM}/ s_i^\text {out}s_j^\text {in}$ for the exponential distribution.

For reconstruction purposes, this model is quite effective as it only requires to know the nodes out- and in-strengths ($s_i^\text {out}$, $s_i^\text {in}$, $\forall \,i$) beside the total number of links ($L^*$). When dealing with production networks, this means having information on the revenues and expenses to and from other firms, as well as some notion of the density of the connections.

#### The limitations of the dcGM

One of the main limitations of the dcGM concerns the inaccurate reconstruction of the mesoscopic features of a network. We will use the following notation to identify groups of nodes (i.e. sectors): $g_i$ is the group to which node *i* belongs (i.e. $i\in g_i$, $\forall \,i$) and we assume that each node belongs to only one group. Hence, $g_i$ and $g_j$ can be the same group even if $i\ne j$ but *i* and *j* belong to the same sector. As an example, let us focus on an inter-firm network and consider the weight between two groups of firms, say $g_r$ and $g_s$, i.e.

4 $$\begin{aligned} s_{g_r\rightarrow g_s}=\sum _{i\in g_r}\sum _{j\in g_s}w_{ij}; \end{aligned}$$

its expected value under the dcGM reads

5 $$\begin{aligned} \langle s_{g_r\rightarrow g_s}\rangle&= \sum _{i\in g_r}\sum _{j\in g_s}\langle w_{ij}\rangle \end{aligned}$$

6 $$\begin{aligned}&=\sum _{i\in g_r}\sum _{j\in g_s}\frac{s_i^\text {out}s_j^\text {in}}{w^\text {tot}}=\frac{s_{g_r}^\text {out}s_{g_s}^\text {in}}{w^\text {tot}},\quad \forall \,g_r\ne g_s \end{aligned}$$

where $s_{g_r}^\text {out} = \sum _{i\in g_r} s_i^\text {out}$ is the total out-strength of group $g_r$ and $s_{g_s}^\text {in} = \sum _{j\in g_s} s_j^\text {in}$ is the total in-strength of group $g_s$.

**Figure 1:**
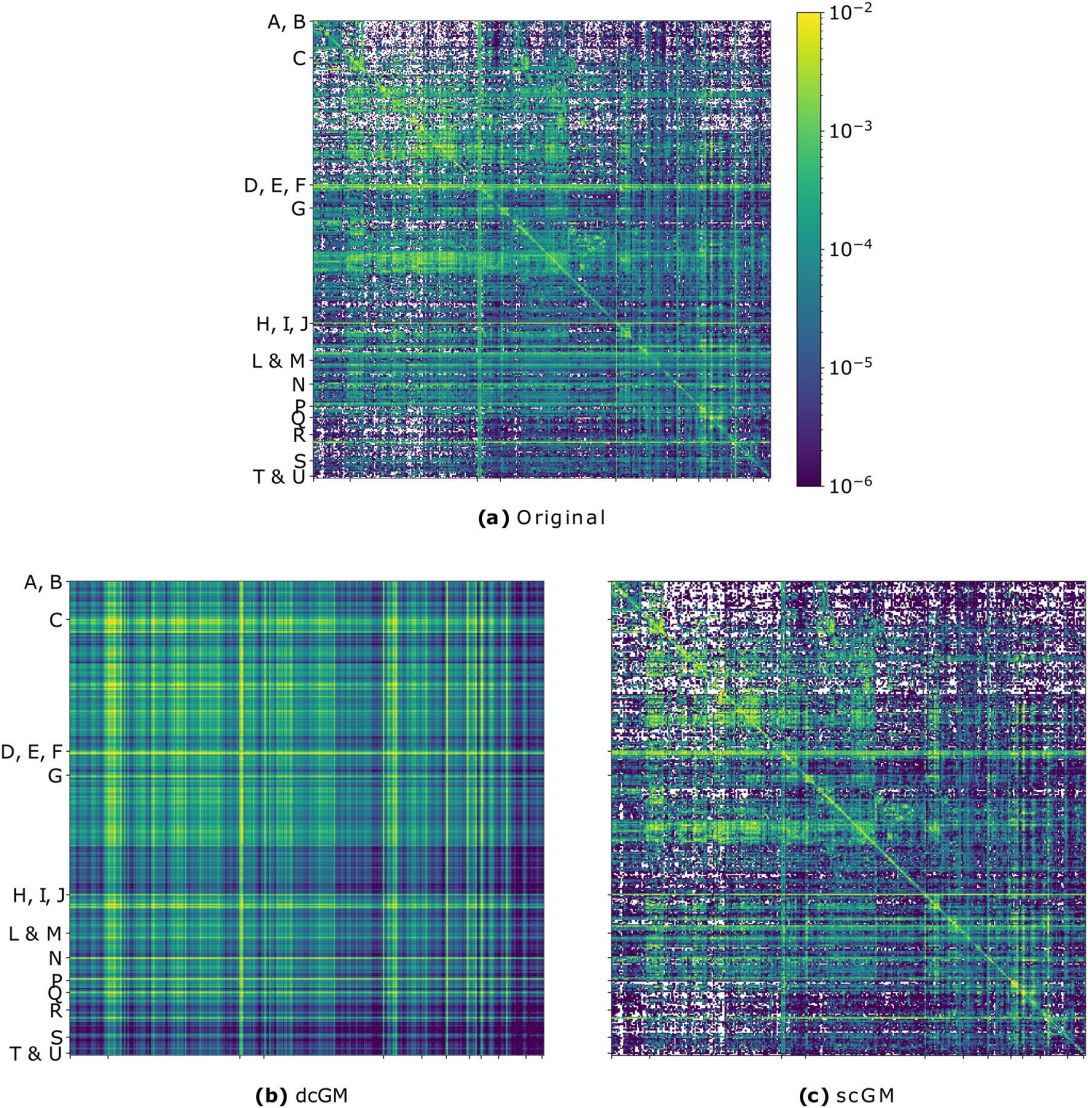
Visualization of the empirical adjacency matrix (**a**) and of the expected adjacency matrix under the dcGM (**b**) and under the scGM (**c**) with rows and columns ordered according to the sector classification of Institution 1. Each row and column in the figure represents a SBI code of 5 digits and the colour represents the empirical/expected density of the non-zero elements of the sub-adjacency matrix representing the firms in the given sectors.

When considering production networks, this will likely differ from the empirical value $s_{g_r\rightarrow g_s}$: the inter-sector flows, in fact, depend on the production technology and it is unlikely that the total sizes of sectors can, by themselves, correctly estimate the input–output dependencies between them. This is also clear from Fig. 1, showing the difference between the inter-sector link-densities expected under the dcGM and their empirical values: the dcGM predicts much more homogeneous rows and columns than the ones we observe in real networks—as an example, consider the predicted high density of connections from some manufacturing sectors towards all sectors (at the top of the C label in Fig. 1b dcGM) whereas there are only very few firms that have those sectors as an input.

### The stripe-corrected Gravity Model

Let us now introduce a model constraining a peculiar kind of mesoscale structures, hereby called ‘stripes’. We do so by defining a new quantity, the in-strength by sector

7 $$\begin{aligned} s_{g_r\rightarrow j}=\sum _{i\in g_r}w_{ij},\quad \forall \,j \end{aligned}$$

representing the list of input quantities by industry of origin $g_r$. This is a good proxy for the amount, by product kind, each node *j* uses to output $s_j^{out}$ units of product. Therefore, it has a clear meaning in terms of economic input–output analysis and Leontief production functions. We can define the *stripe-corrected Gravity Model* (scGM) as a two step procedure analogous to the dcGM one, with the parameter $\beta_{ij}$ chosen to ensure that

8 $$\begin{aligned} \langle w_{ij} | a_{ij} = 1 \rangle = \frac{s_i^\text {out}s_{g_i\rightarrow j}}{w_{g_i}^\text {tot}\cdot p_{ij}^\text {scGM}},\quad \forall \,i\ne j \end{aligned}$$

where

9 $$\begin{aligned} p_{ij}^\text {scGM}=\frac{zs_i^\text {out}s_{g_i\rightarrow j}}{1+zs_i^\text {out}s_{g_i\rightarrow j}},\quad \forall \,i\ne j \end{aligned}$$

and that we have normalized with the total outgoing strength of sector $g_i$, i.e.

10 $$\begin{aligned} \sum _{k\in g_i}s_k^\text {out}=\sum _{k\in g_i}\sum _j w_{kj}=\sum_j s_{g_i\rightarrow j}\equiv w^\text {tot}_{g_i},\quad \forall \,g_i; \end{aligned}$$

notice that the first and the last sum simply account for the same addenda, just sorting them differently. While the relationship $\sum_i s_i^\text {out} = \sum_j s_j^\text{in} = w^\text{tot}$ ensures the dcGM to be normalized in such a way to reproduce out-strengths and in-strengths, Eq. (10) now ensures the scGM to be normalized in such a way to reproduce out-strengths and sectorial in-strengths. In fact,

11 $$\begin{aligned} \langle s_i^\text {out}\rangle&=\sum _j\langle w_{ij}\rangle =\sum _j\frac{s_i^\text {out}s_{g_i\rightarrow j}}{w_{g_i}^\text {tot}}=s_i^\text {out}\frac{\sum _js_{g_i\rightarrow j}}{w_{g_i}^\text {tot}}=s_i^\text {out},\quad \forall \,i, \end{aligned}$$

12 $$\begin{aligned} \langle s_{g_i\rightarrow j}\rangle&=\sum _{k\in g_i}\langle w_{kj}\rangle =\sum _{k\in g_i}\frac{s_k^\text {out}s_{g_i\rightarrow j}}{w_{g_i}^\text {tot}}=s_{g_i\rightarrow j}\frac{\sum _{k\in g_i}s_k^\text {out}}{w_{g_i}^\text {tot}}= s_{g_i\rightarrow j},\quad \forall \,g_i,\,j; \end{aligned}$$

removing self-contributions, instead, leads to the slightly distorted relationships $\langle s_i^\text {out}\rangle =s_i^\text {out}-\frac{s_i^\text {out} s_{g_i\rightarrow i}}{w_{g_i}^\text {tot}}$, $\forall \,i$ and $\langle s_{g_j\rightarrow j}\rangle =s_{g_j\rightarrow j}-\frac{s_j^\text {out}s_{g_j\rightarrow j}}{w^\text {tot}_{g_j}}$, $\forall \,j\in g_j$. Furthermore,

13 $$\begin{aligned} \langle s_j^\text {in}\rangle&=\sum _i\langle w_{ij}\rangle =\sum _i\frac{s_i^\text {out}s_{g_i\rightarrow j}}{w_{g_i}^\text {tot}}=\sum _{g_i}\sum _{k\in g_i}\frac{s_k^\text {out}s_{g_i\rightarrow j}}{w_{g_i}^\text {tot}}=\sum _{g_i}s_{g_i\rightarrow j}=s_j^\text {in},\quad \forall \,j, \end{aligned}$$

14 $$\begin{aligned} \langle s_{g_i}^\text {out}\rangle&=\sum _{k\in g_i}\sum _j\langle w_{kj}\rangle =\sum _{k\in g_i}\sum _j\frac{s_k^\text {out}s_{g_i\rightarrow j}}{w_{g_i}^\text {tot}}=\sum _{k\in g_i}s_k^\text {out}\frac{\sum _j s_{g_i\rightarrow j}}{w_{g_i}^\text {tot}}=\sum _{k\in g_i}s_k^\text {out}=s_{g_i}^\text {out},\quad \forall \,g_i; \end{aligned}$$

where again, removing self-contributions introduces the slightly distorted relationships $\langle s_j^\text {in}\rangle =s_j^{in}-\frac{s_j^\text {out}s_{g_j\rightarrow j}}{w^\text {tot}_{g_j}}$, $\forall \,j$ and $\langle s_{g_i}^\text {out}\rangle =s_{g_i}^\text {out}-\sum _{k\in g_i} \frac{s_k^\text {out} s_{g_i\rightarrow i}}{w_{g_i}^\text {tot}}$, $\forall \,g_i$. Differently from the dcGM, in this model the diagonal element $w_{ii}$ can be zero also ‘naturally’: this happens if $s_{g_i\rightarrow i}=0$, $\forall \,i$, that is, if there are no links between nodes in the same sector. In case we could distinguish companies exactly by the product they produce this would likely be the case; on the other hand, if the above is not true, the weight some of the nodes must redistribute might be larger than for the dcGM case as the denominator is now the total strength of the sector and not of the whole graph. The only parameter, *z*, can be again found by solving the equation

15 $$\begin{aligned} \langle L\rangle =\sum _i\sum _{j(\ne i)}\frac{zs_i^\text {out}s_{g_i\rightarrow j}}{1+zs_i^\text {out}s_{g_i\rightarrow j}}=L^*; \end{aligned}$$

importantly, since *z* is a global parameter affecting all the $p_{ij}$ the same way, errors accompanying the evaluation of $L^*$ would not significantly affect the relative probabilities of the nodes. Indeed, if $p_{ij}>p_{sr}$, this will be still true no matter the *z* value (except for the two extremes, i.e. $z=0$, which gives an empty graph, and $z\rightarrow \infty$, which asymptotically results in a fully connected graph).

One issue that may arise is related to the number of outgoing links from each sector $g_i$, i.e.

16 $$\begin{aligned} L_{g_i}\equiv \sum _{k\in g_i}\sum _{j} a_{kj},\quad \forall \,g_i; \end{aligned}$$

clearly, in Eq. (15) we are constraining $\sum _{g_i} L_{g_i}$ but we have no guarantees over the single $L_{g_i}$: therefore, should we have access to the coefficients $L_{g_i}$, $\forall \,g_i$ we could account for the different densities in the sectors by defining a multi-*z* variant of the model. The only difference would consist in the definition of the probabilities $p_{ij}^\text {scGM}$ that, now, would read

17 $$\begin{aligned} p_{ij}^\text {scGM}=\frac{z_{g_i}s_i^\text {out}s_{g_i\rightarrow j}}{1+z_{g_i}s_i^\text {out}s_{g_i\rightarrow j}},\quad \forall \,i\ne j; \end{aligned}$$

the same quantities are preserved in the multi-*z* variant and the parameters $z_{g_i}$, $\forall \,g_i$ can be found by solving the equations

18 $$\begin{aligned} \langle L_{g_i}\rangle =\sum _{k\in g_i}\sum _j\frac{z_{g_i}s_k^\text {out} s_{g_i\rightarrow j}}{1+z_{g_i}s_k^\text {out}s_{g_i\rightarrow j}}=L_{g_i}^*,\quad \forall \,g_i. \end{aligned}$$

The main advantage of the stripe-corrected model is that of introducing a strong restriction on the number of available links. In fact, according to the dcGM, the probability of observing a link between *i* and *j* cannot be zero unless either $s_i^\text {out}$ or $s_j^\text {in}$ is zero—a property that is also shared by models constraining block structures^30^: the Stochastic Block Model (SBM), for example, preserves the number of links between groups; hence, it can predict the existence of a link between a firm in sector A and a firm in sector B as long as there is (at least) one company in A with a connection to (at least) one company in B. As a sufficiently-detailed sector classification would induce a significant sparsity in the sectors, using only the inter-block density would lead to an unjustified homogenisation of the neighbours of each node: to capture the desired behaviour, we need a model ensuring that if a company does not buy any product from a given sector, no connection can exist between that company and any other in the said sector. Such a possibility, that translates into the request of observing many zero $s_{g_i\rightarrow j}$ terms, is successfully handled by the scGM and ensures it to perform better than the dcGM in reproducing the true zeros of the adjacency matrix (for a more detailed discussion about the relationship between the scGM and block models^30,31^ see the SI).

### Data

**Table 1:** Number of distinct sectors by hierarchical level used in classification codes.

Level	SBI rule	NAICS rule	ABN	ING
1	Area	2 digits	19	23
2	2 digits	3 digits	82	89
3	3 digits	4 digits	253	303
4	4 digits	5 digits	567	642
5	All digits	All (6) digits	888	953

We have validated our methodology on the properties of two similar datasets, made available for this research by ABN AMRO Bank N.V. (ABN) and ING Bank N.V. (ING). The information was not shared by the two banks but rather the analysis was conducted in parallel such that no sensitive information was transferred between the two institutions. Only the anonymous results have been made available for the purpose of writing the present paper.

The raw data consists of a table of money flows to and from the accounts of the commercial clients of the banks from 2018 (January for ABN and October for ING) to the present date. Our analysis will therefore focus on the year 2019, for which the data is fully available and during which there have not been any extreme events that might affect the result of our analysis. This data is primarily constituted of SEPA transactions which are by far the most common type of cashless payment for these accounts. Given that we see on the clients accounts movements coming and going to non-client accounts, it would be possible for us to include non-clients as nodes in our network. However, the available information is severely limited and we were often unable to distinguish between accounts belonging to a firm or a private individual; hence, in order to avoid possible biases introduced by this discrepancy, we have limited ourselves to build our networks by just considering client-to-client transactions.

We define the networks as directed, the direction of the flow being opposite to the direction of the money transfer. We further assign each node to a sector using its industrial classification code. These codes are hierarchical in nature allowing us to define five hierarchical levels of sector definitions with level five being the most specific (see table 1 for a summary of the various hierarchical levels in both datasets). Hence in our networks, an edge from firm *i* in Sector A to firm *j* in Sector B with weight 100, will represent the sale of a product of type A by firm *i* to firm *j* for the value of 100 Euros. After this construction, the two networks, one for ING and one for ABN clients, have in the order of $10^5$ nodes and $10^6$ links each (see the Supplementary Information section S.6 for further discussion on the data).

## Results

### Sector information

To quantitatively test the added value of introducing information about sectors, let us compare the dcGM and the scGM likelihood of observing the binary component of our empirical graphs. The term ‘likelihood’, here, refers to the one of the adjacency matrix, given that the main difference between the dcGM and the scGM lies in the preserved binary structure.

**Figure 2:**
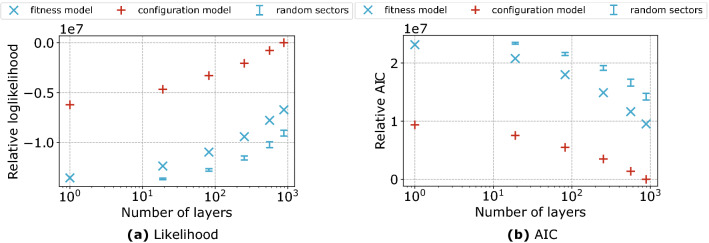
Difference in log likelihood (**a**) and AIC (**b**) of the fitted models for an increasing number of sectors (layers) for the data of Institution 1. The fitness model refers to the dcGM when only one layer exists—in this case, in fact, the scGM and the dcGM coincide—and to the scGM otherwise. The results are given with respect to the best performing model. The error bars show the intervals of log likelihoods for the fitted scGM models where the sector labels have been randomized.

Let us consider the formulation of the scGM provided in Eq. (18) (in this case, the number of free parameters in the model is equal to the number of distinct sector labels): in Fig. 2 we plot the likelihood of the fitted models as a function of the number of distinct sector labels given to the scGM, hereby called ‘layers’—the name comes from the fact that we can interpret the edges as existing on separate product layers of a multi-layer graph. In order to get a correct comparison with the dcGM, the DCM is applied to each layer independently (see Supplementary Information section S.2 for further information about the relationship between the two models). As Fig. 2 shows, the scGM outperforms the dcGM on both networks, at all hierarchical levels: the increase of the log-likelihood, in fact, more than offsets the increase of number of parameters, as the Akaike Information Criterion (AIC) reveals. Notice that the likelihood increase caused by a finer resolution in sector classification is comparable to the likelihood increase caused by replacing the fitness model with the DCM, i.e. a model whose full specification requires the knowledge of the true degree sequence.

Although the AIC allows us to rule out the possibility of over-fitting by increasing the number of free parameters, we also want to exclude the possibility that the introduction of any stripe-like meso-structure could lead to the same bias. To this end, we have compared the log-likelihood and the AIC of our scGM with a variant of it defined by randomly permuting the sector label of all firms, thus keeping the same number of sectors and sector densities. After the labels are permuted, the $s_{g_j \rightarrow i}$ values are recomputed to ensure consistency: as such, this variant is equal to the scGM applied to a new network with the same connections but a different sector structure. We have performed one hundred permutations and fitted the scGM to the graphs with permuted labels at the various hierarchical levels. The results are shown in Fig. 2 as confidence interval and clearly demonstrate that the real sector dependencies carry much more information than the random labels.

To understand why this may be the case, one can consider the relationship between the in-strength by sector and the entropy of the scGM. Every zero entry in the in-strength by sector sequence of a node sets the probability of connections coming from that group to zero. This restricts the number of configurations of the system thus reducing the overall entropy of the ensemble. It is, then, clear that the entropy of this model is maximized when no entry of $s_{g_i\rightarrow j}$ is zero. Empirical relationships between firms and sectors tends to be very sparse, with firms having their incoming connections concentrated into few groups; however, these patterns are destroyed by permuting the labels. From the model point of view, the permutation of the labels increases the number of non-zero $s_{g_i\rightarrow j}$ terms, hence increasing the entropy of the ensemble and lowering the performance of the model. A secondary way in which permutations can affect the quality of fit is by disrupting the correlation that exists between the size of the companies and the sector they belong to. In the scGM, it is the size of a firm relative to its sector that determines the probability of establishing connections: as such, after the randomization a firm that was comparatively large can become small in the new group and vice-versa.

The fact that the true labels of the nodes induce a sparse $s_{g_i\rightarrow j}$ structure can be visualised by looking at the density of connections between submatrices of the adjacency matrix: Fig. 1 clearly shows that the scGM is able to reproduce the sparse structure of the sector relations. Notice that the white spaces indicate zero probability of connection and are going to be significantly reduced in the case of permuted labels. From Fig. 1, we can also appreciate the improvement that the scGM achieves with respect to the dcGM in correctly reproducing the mesoscale structure of the inter-sector relations.

**Figure 3:**
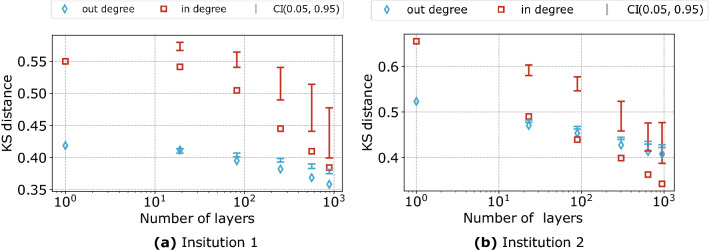
Kolmogorov–Smirnoff distance between the degree distribution induced by the model and the empirical one for institution 1 (**a**) and Institution 2 (**b**). The error bars show the intervals of KS distances for the fitted scGM models with the randomized sector labels. Notice that, in all cases, the hypothesis that the two distributions coincide is rejected.

### Entropy of the system

Given that the likelihood in ERG models is equal to the negative entropy of the ensemble (see section S.2 in the Supplementary Information) we can also interpret the likelihood increasing with the sector resolution in Fig. 2 as a reduction in the entropy of the system, i.e. in the number of alternative network configurations that preserve the same numbers of in- and out-neighbours, along with their input and output flows by sector. Specifically, the relationship between entropy and likelihood is given by

19 $$\begin{aligned} S(\vec {\tilde{\alpha }}, \vec {\tilde{\beta }}) = \sum _{i=1}^N [\tilde{\alpha }_i \langle k^{out}_i \rangle +\tilde{\beta }_i \langle k^{in}_i \rangle ] + \ln Z(\vec {\tilde{\alpha }},\vec {\tilde{\beta }}) = - \mathscr {L}(G^*|\vec {\tilde{\alpha }}, \vec {\tilde{\beta }}) \end{aligned}$$

where $\mathscr {L}(G^*|\vec {\tilde{\alpha }}, \vec {\tilde{\beta }})$ is the log-likelihood of the fitted model, $\vec {\tilde{\alpha }}, \vec {\tilde{\beta }}$ are the parameters due to the fitness ansatz, and $S(\vec {\tilde{\alpha }}, \vec {\tilde{\beta }})$ is the entropy of a Directed Configuration Model (DCM) with expected degree sequences $\vec {k}^{in}$ and $\vec {k}^{out}$. From Eq. (19) we can see that the difference in entropy between the two models is related to the difference between real and expected degrees.

For each model, the reduction in entropy with increasing sector resolution indicates that considering a single aggregate sector or good leads to an over-estimation of the number of alternative configurations and, therefore, of the rewiring capability of the system. Finer product resolution improves our estimate of the entropy by disallowing connection between firms and the sectors that do not belong to their input. The entropy decrease with respect to the dcGM—allowing interactions between firms in all sectors—is unavoidable, hence not surprising, for any model that introduces restrictions on the wiring possibilities. The permutation experiment confirms that the true industry classification reduces the entropy of the ensemble by a larger degree than any random or arbitrary classification.

This result is important, as it provides ground for the scGM to be used as a realistic generative model of inter-firm networks subject to the observed production constraints and, by considering only viable rewiring opportunities for firms, as an estimator via its entropy of the resilience of the system to shocks that may force it out of its current microscopic network configuration. Like entropy in statistical physics, that estimates the number of alternative microscopic configurations that a large system can visit while remaining in the same macroscopic equilibrium state, the entropy of a network ensemble enumerates the alternative network configurations that the system can explore while remaining in the same aggregate production state, i.e. without firms being forced to change any of their aggregate input and output constraints by sector.

**Figure 4:**
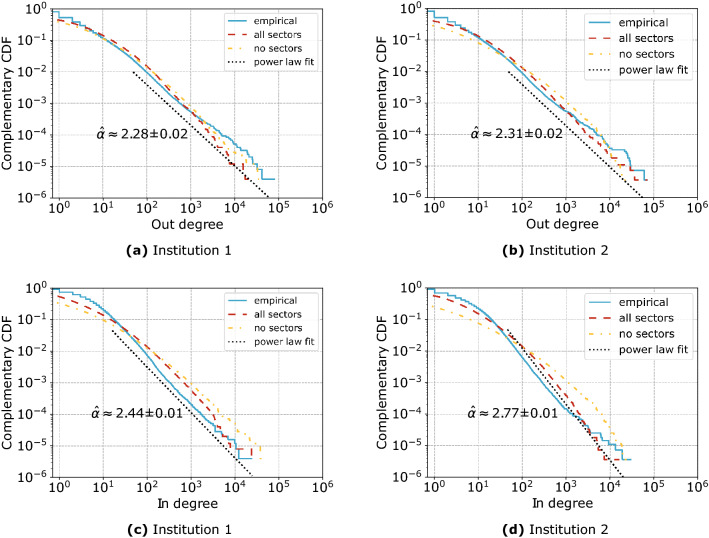
Complementary cumulative distributions of the out- and in-degrees for the network of Institution 1 (**a**,**c**) and Institution 2 (**b**,**d**).

### Structural properties

We can further assess the correspondence between model and empirical networks by looking at several structural properties and macroscopic trends. In Fig. 4 we notice that both the dcGM (no sectors) and the scGM applied together with the level 5 sector definitions (all sectors) accurately reproduce the cumulative distributions of the out- and in-degrees of the empirical networks with only minor differences (e.g. the scGM performs better in reproducing the distribution of in-degrees, especially in the extremes). We also test which distribution, between exponential, log-normal and power-law, best fits the data using a likelihood ratio test. In all cases the empirical distribution is fat-tailed with the hypothesis of following an exponential distribution being rejected with a *p* value that is less than $10^{-6}$. We find mixed support for power-law and log-normal distributions, with a slight preference for the power-law: in Fig. 4a power-law (with *p* value 0.10), in Fig. 4b power-law (0.01), in Fig. 4c log-normal ($<0.001$), and in Fig. 4d power-law (0.04). The range of exponents of the power-law distributions that we find ($\hat{\alpha } \in [2.28, 2.77]$) is in line with what has been found for other supply networks: the power-law exponents estimated for the Japanese network are $\alpha =2.26$ and $\alpha =2.35$, respectively for the out- and in-degree distributions^32^; similarly, the undirected degree distribution of the Hungarian supply network obeys a power-law with $\alpha =2.40$^17^.

We, then, quantitatively assess the improvement in the quality of fit of the degree distributions achieved by introducing sector labels, by employing the Kolmogorov–Smirnov test. Similarly to what discussed in section “Sector information”, we also perform the test on the models with permuted labels. From the results in Fig. 3 we again find a significant improvement in the quality of fit for increasing sector information. We notice, as expected, that the effect is larger on the in-degrees since it is the in-strength that is being divided by sector.

**Figure 5:**
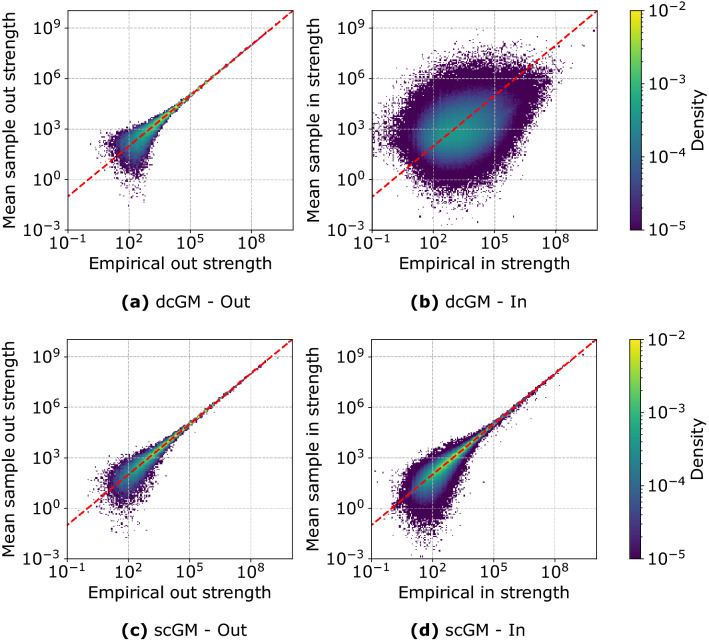
Empirical vs mean node strength by sector computed over 100 samples drawn from the dcGM (**a**, **b**) and scGM (**c**, **d**) ensembles for the network of Institution 1.

So far, we have shown only properties that are not defined to be sector specific. However, the main difference between the models lies precisely in the ability to preserve sector information. We can visualize this difference by looking at the empirical strength by sector and at the average one, computed over one hundred samples. The comparison is shown in Fig. 5: the plots reveal three important aspects about the way the dcGM and the scGM work. First, there is almost no difference in the accuracy of the out-strength: this is a consequence of the way the scGM is formulated, with the out-strength being always the total one, rather than the sector-specific one, such that each node will have out-strength by sector equal to zero, for all sector except the one they belong to.

A second observation concerns the different variances characterizing low and high values of strength. This spread is a consequence of limited sampling: indeed the strength is preserved by definition as shown in Eqs. (11) and (12). However, this is true only on average and not for each specific sampled configuration. The sample variance for these measures is larger for low strength values because the probability of observing the corresponding link is lower. This means that more often no link will be sampled; hence, the expected value of the weight, given that the link is sampled, will be further from the expected value as can be deduced from the presence of $p_{ij}$ at the denominator of Eq. (8). These two mechanisms cause the higher variance that we observe.

Finally, and most importantly, the way the in-strength is distributed by sector in the dcGM does not resemble the information in the original graph. Notice that we are only showing the values when the empirical in-strength is non-zero. The dcGM, however, almost always assigns a positive value to it even if, empirically, it is zero. This is an important point because it tells us a key weakness of the dcGM that motivates the formulation of the scGM: the dcGM is unable to correctly reconstruct the neighbourhood of a node in terms of sectors.

**Figure 6:**
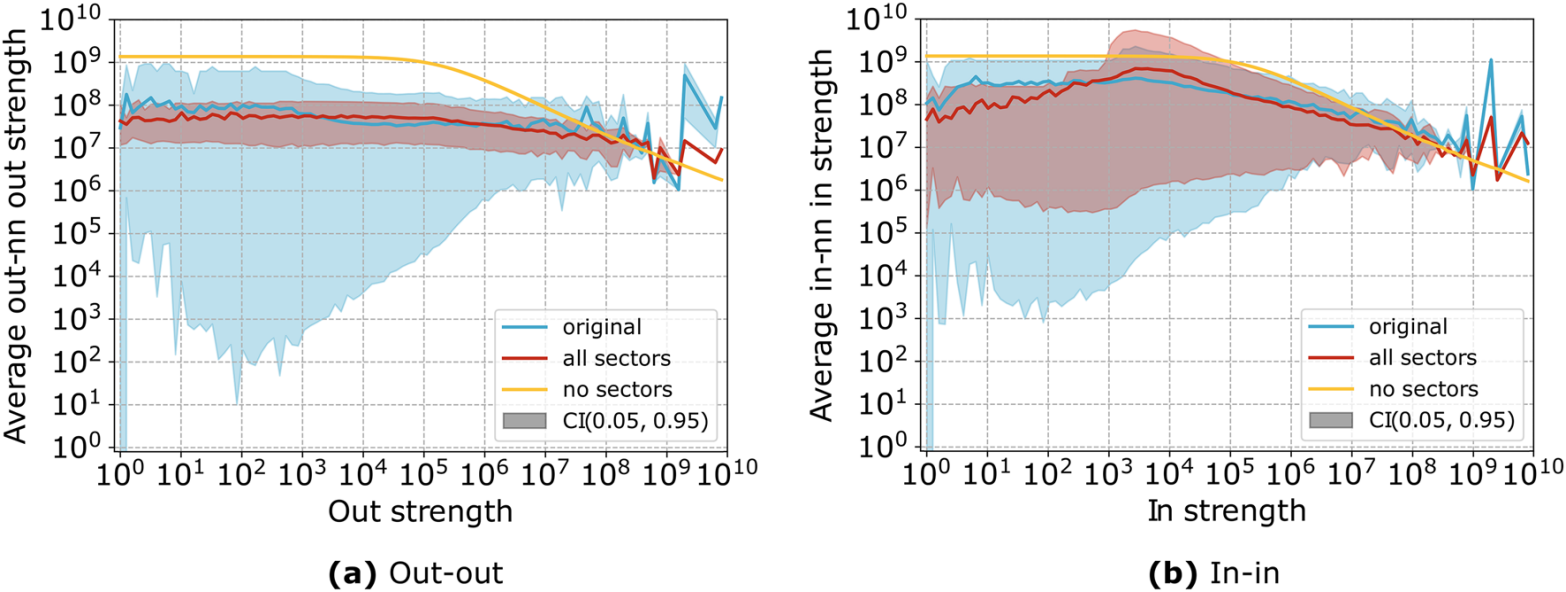
Average nearest out-neighbour out-strength vs node out-strength (**a**) and average nearest in-neighbour in-strength vs node in-strength (**b**) for Institution 1. The full lines are computed as the average over all firms (x-axis has been logarithmically binned). The shaded area represents the interval between the 5th and 95th percentile measured over the same bins.

We further explore the quality of reconstruction of the neighbourhood of each node by looking at the average nearest neighbours strength. In Fig. 6 we show the pattern for the out-out and in-in average nearest neighbours strength: we can clearly observe that the scGM (red) follows more closely the empirical trend (blue) than the dcGM (yellow). Furthermore, the narrow confidence interval for the dcGM is due to the fact that the dcGM assigns exactly the same neighbourhood to nodes with the same strength no matter what sector they belong to. This is clearly unrealistic and proves that the dcGM is not suitable when applied to production networks.

## Discussion

Production networks are extremely important to understand the response of an economic system to shocks but the scarcity of relevant data makes analyses like these difficult. In this work we proposed a methodology that, based on limited and aggregate information on firms revenues and expenses by industry, can accurately reproduce key structural properties of the original network. We have shown that, by introducing sector-specific information, we can better reproduce macroscopic properties such as the degrees and the average nearest neighbours strength of each firm separately. It is important to remark that being able to estimate these properties is key in any application concerning production networks. For instance, capturing the correct statistical behaviour of the average nearest in-neighbour in-strength by sector leads to a realistic estimation of sector composition of two-step interactions in a supply chain. Of course, we cannot expect the scGM to reproduce each neighbourhood exactly given the extremely limited information used to generate the ensemble; nevertheless, it is very encouraging that such a simple methodology can reproduce the empirical patterns so accurately.

As an added value with respect to other modelling strategies, our maximum-entropy approach can estimate the underlying rewiring capabilities of the system. Ignoring the sector specificity of firms leads to an overestimation of the alternative input- and output-preserving configurations of the inter-firm network: in practical applications, this would result in a systematic overestimation of the resilience of the system. As the scGM is able to correctly encode sector-specific information, it is a good candidate to be used for generating realistic probability distributions for inter-firm networks subject to fine production constraints and to estimate the entropy of the ensemble as a proxy for the rewiring capabilities of the system. Indeed the entropy calculated from our model proxies the number of alternative network configurations that are still compatible with the observed supply and demand constraints by sector for each firm, thus leaving the entire system in its current production state, much like entropy in statistical mechanics enumerates the alternative microscopic configurations that a large system can potentially explore along its dynamical trajectory while remaining in the same equilibrium macrostate.

Differently from existing maximum entropy approaches, the scGM requires, in addition to the knowledge of the total revenue of each firm ($s_i^\text {out}$) and the total number of links of the graph ($L^*$), the expenses broken down by sector ($s_{g_j \rightarrow i}$). If the $s_{g_j \rightarrow i}$ were not known, they should be imputed using other data: for example, if more aggregate information were available, such as the input–output tables, one could use the flows between sectors and compute $s_{g_j \rightarrow i}$ by assuming each firm to have the same ratios between inputs as the whole sector. In our opinion, however, this would undermine the formulation of the scGM which allows the heterogeneity between firms in the same sector to be preserved: therefore, it would be necessary to have access to some other information allowing the values $s_{g_j \rightarrow i}$ to be correctly estimated for each firm. If, instead, the density were not known, it could be left as a free parameter to inspect how the properties of the network would change as the graph were made sparser or denser.

The analytical tractability of this model allows us to find many expected properties over the ensemble without having to generate samples. For all other applications, such as shock propagation exercises, samples can be generated efficiently and without bias.

## Supplementary Information

Supplementary Information.

## Data Availability

The datasets on transactions used in the paper are highly confidential and cannot be made public.

## Code availability

Code is available upon request.
