# Supplementary material for: Reconstructing firm-level interactions in the Dutch input–output network from production constraints
Source: Sci Rep. 2022 Jul 13;12:11847. doi: 10.1038/s41598-022-13996-3 (PMC9277606; doi:10.1038/s41598-022-13996-3)
Supplement: Supplementary file 1 — Supplementary Information. [file 41598_2022_13996_MOESM1_ESM.pdf]

Supplementary Information

Reconstructing firm-level interactions in the Dutch input-output network from production constraints

Leonardo Niccolò Ialongo[1,2,3,*], Camille de Valk[4,5], Emiliano Marchese[1,3], Fabian Jansen[5], Hicham Zmarrou[3], Tiziano Squartini[1,6], and Diego Garlaschelli[1,4]

[1]IMT School for Advanced Studies, Lucca, 55100, Italy
[2]Scuola Normale Superiore, Pisa, 56126, Italy
[3]ABN AMRO Bank N.V., Amsterdam, 1082 PP, The Netherlands
[4]University of Leiden, Lorentz Institute for Theoretical Physics (LION), Leiden, 2333 CA, The Netherlands
[5]ING Bank N.V., Amsterdam, 1102 CT, The Netherlands
[6]University of Amsterdam, Institute for Advanced Study (IAS), Amsterdam, 1012 GC, The Netherlands
[*]leonardo.ialongo@sns.it

S.1 Multiproduct generalization

There is a clear relationship between the formulation of the scGM and the firms production functions that is best undestood upon looking at the multi-product generalization of the model presented. We define Γ to be the set of all goods available in our economy and $\gamma \in \Gamma$ to be one such product. Our network is now multi-layered with the generic entry of the directed, weighted adjacency matrix w_{ij}^{γ} corresponding to the amount of goods of type γ sold by i and bought by j. The out- and in-strengths for each node can now be defined product-wise as follows:

$$s_{i,\text{out}}^{\gamma} = \sum_{j(\neq i)} w_{ij}^{\gamma}, \quad \forall i, \tag{1}$$

$$s_{i,\text{in}}^{\gamma} = \sum_{j(\neq i)} w_{ji}^{\gamma}, \quad \forall i. \tag{2}$$

We can now reformulate the two steps of the scGM for sampling edges and their weights: the probability of observing a link between nodes i and j of type γ is given by

$$p_{ij}^{\gamma} = \frac{z s_{i,\text{out}}^{\gamma} s_{j,\text{in}}^{\gamma}}{1 + z s_{i,\text{out}}^{\gamma} s_{j,\text{in}}^{\gamma}}, \quad \forall i \neq j \tag{3}$$

and the weights can be assigned via the recipe

$$\langle w_{ij}^{\gamma} | a_{ij}^{\gamma} = 1 \rangle = \frac{z s_{i,\text{out}}^{\gamma} s_{j,\text{in}}^{\gamma}}{w_{\gamma}^{\text{tot}} \cdot p_{ij}^{\gamma}}, \quad \forall i \neq j \tag{4}$$

where $w_{\gamma}^{\text{tot}} = \sum_i \sum_{j(\neq i)} w_{ij}^{\gamma}$. This model can be fitted to data by using either the total density of connections to find z or by letting the density depend on the product layer. In the latter case z is replaced by z^{γ} and it is found by letting

$$\langle L^{\gamma} \rangle = \sum_i \sum_{j(\neq i)} p_{ij}^{\gamma} = \sum_i \sum_{j(\neq i)} a_{ij}^{\gamma\,*} = (L^{\gamma})^{*}. \tag{5}$$

This model might appear different from the scGM given that both the out- and in-strengths are now product-wise. However, under the assumption that each firm produces only one good that is the same for each business in a given industry, the generalization above and the model presented in the main text coincide. Under this restriction, then the product γ coincides with the industry producing it g_i, giving $\gamma = g_i$. It then follows that $s_{i,\text{out}}^{\gamma} = s_i^{\text{out}}$ for $\gamma = g_i$ and zero otherwise and, similarly, $s_{j,\text{in}}^{\gamma} = s_{g_i \to j}$ given $\gamma = g_i$. The generalization above also clarifies that input and output strengths are not *per se* treated differently; it is rather the choice of the proxy used to identify goods (in our case, the sector classification) that causes only one out-strength to be different from zero.

S.2 Entropy and likelihood in Exponential Random Graphs

Both the dcGM and the scGM derive from the general family of constrained, maximum-entropy graph ensembles. We recap here some of the important properties of this group of models.

Maximum-entropy graph ensembles are a result of the approach proposed by Jaynes to maximize Shannon entropy subject to constraints[1]. This can be achieved by maximizing the functional

$$\mathscr{L}[P] = S[P] - \lambda_0 \left[\sum_{G\in\mathscr{G}} P(G) - 1\right] - \sum_{m=1}^{M} \lambda_m \left[\sum_{G\in\mathscr{G}} P(G) C_m[G] - \langle C_m\rangle\right] \tag{6}$$

where $S[P]$ is Shannon entropy, defined by $S = \sum_{G\in\mathscr{G}} -P(G)\ln P(G)$ and $\{C_m[G]\}_{m=1}^{M}$ is the set of constraints encoding known information about the ensemble. Solving the maximization problem yields the general form of Exponential Random Graphs (ERG)[2]:

$$P(G|\vec{\lambda}) = \frac{e^{-\sum_{m=1}^{M}\lambda_m C_m[G]}}{Z(\vec{\lambda})} \tag{7}$$

where $Z(\vec{\lambda})$ is the partition function defined as $Z(\vec{\lambda}) = \sum_{G\in\mathscr{G}} e^{-\sum_{m=1}^{M}\lambda_m C_m[G]}$. Given a real graph G^* we would like to find the set of Lagrange multipliers $\{\lambda_m\}_{m=1}^{M}$ that maximize the log-likelihood

$$\mathscr{L}(G^*|\vec{\lambda}) = \ln P(G^*|\vec{\lambda}) = -\sum_{m=1}^{M} \lambda_m C_m[G^*] - \ln Z(\vec{\lambda}); \tag{8}$$

it can be shown that the maximum of the log-likelihood is found for the values $\vec{\lambda}^*$ ensuring that the expected value of the constraints matches their empirical value, i.e. $\langle C_m\rangle = C_m[G^*] = C_m^*$. Since the entropy of ERGs is given by

$$S(\vec{\lambda}) = \sum_{G\in\mathscr{G}} -P(G|\vec{\lambda})\ln P(G|\vec{\lambda}) \tag{9}$$

$$= -\sum_{G\in\mathscr{G}} P(G|\vec{\lambda})\left(-\sum_{m=1}^{M}\lambda_m C_m[G] - \ln Z(\vec{\lambda})\right) \tag{10}$$

$$= \sum_{m=1}^{M}\lambda_m \sum_{G\in\mathscr{G}} P(G|\vec{\lambda}) C_m[G] + \ln Z(\vec{\lambda}) \sum_{G\in\mathscr{G}} P(G|\vec{\lambda}) \tag{11}$$

$$= \sum_{m=1}^{M}\lambda_m \langle C_m\rangle + \ln Z(\vec{\lambda}) \tag{12}$$

the maximum-likelihood estimation $\vec{\lambda}^*$ of $\vec{\lambda}$, guaranteeing that $\langle C_m\rangle = C_m^*$, allows us to write

$$S(\vec{\lambda}^*) = \sum_{m=1}^{M}\lambda_m^* C_m^* + \ln Z(\vec{\lambda}^*) = -\mathscr{L}(G^*|\vec{\lambda}^*). \tag{13}$$

Therefore, the entropy of an ERG model is equal to minus the log-likelihood of observing any graph whose constrained quantities match the ensemble expectations. In practice, this tells us that given two ERG models, fitted using the maximum-likelihood principle, the one with higher likelihood will necessarily have a lower entropy.

It is important to recall here that the first step of both the dcGM and the scGM is derived from the Directed Configuration Model (DCM)[3] which is an ERG model for directed graphs. The DCM is found by constraining the out- and in-degrees $\vec{k}^{in}(G^*), \vec{k}^{out}(G^*)$ of a given graph G^*. This results, after maximizing the likelihood, in the probability distribution (detailed derivation can be found in[4])

$$P(G|\vec{\alpha}^*, \vec{\beta}^*) = \frac{e^{-\sum_i [\alpha_i^* k_i^{\text{out}}(G) + \beta_i^* k_i^{\text{in}}(G)]}}{Z(\vec{\alpha}^*, \vec{\beta}^*)} = \prod_i \prod_{j(\neq i)} p_{ij}^{a_{ij}} (1-p_{ij})^{1-a_{ij}} \tag{14}$$

with

$$p_{ij} = \frac{e^{-\alpha_i^* - \beta_j^*}}{1 + e^{-\alpha_i^* - \beta_j^*}}, \tag{15}$$

where the $2N$ Lagrange multipliers $\vec{\alpha}, \vec{\beta}$ have been set to their maximum-likelihood values $\vec{\alpha}^*, \vec{\beta}^*$ ensuring that the expected values of all the out- and in-degrees equal the empirical values $\vec{k}^{in}(G^*), \vec{k}^{out}(G^*)$ *exactly*, i.e.

$$\langle k_i^{in} \rangle = \sum_{j(\neq i)} \frac{e^{-\alpha_j^* - \beta_i^*}}{1 + e^{-\alpha_j^* - \beta_i^*}} = k_i^{in}(G^*), \quad \forall i, \tag{16}$$

$$\langle k_i^{out} \rangle = \sum_{j(\neq i)} \frac{e^{-\alpha_i^* - \beta_j^*}}{1 + e^{-\alpha_i^* - \beta_j^*}} = k_i^{out}(G^*), \quad \forall i. \tag{17}$$

Notice that finding the values $\vec{\alpha}^*, \vec{\beta}^*$ requires solving the $2N$ nonlinear coupled equations above.

The dcGM follows from the so-called fitness ansatz, formulated in[3], positing that $e^{-\alpha_i^*} \approx \sqrt{z} s_i^{\text{out}}$ and $e^{-\beta_j^*} \approx \sqrt{z} s_j^{\text{in}}$, with z being the only remaining free parameter, which can be used to fit the empirical density of the graph G^*. Substituting these values into equation (15) we obtain the dcGM

$$p_{ij} \approx p_{ij}^{\text{dcGM}} = \frac{z^* s_i^{\text{out}} s_j^{\text{in}}}{1 + z^* s_i^{\text{out}} s_j^{\text{in}}} \tag{18}$$

where z^* is such that the expected link density equals the empirical one, i.e. the expected total number of links $\langle L \rangle$ equals the empirical total number of links $L(G^*)$ of the real graph G^*:

$$\langle L \rangle^{\text{dcGM}} = \sum_i \sum_{j(\neq i)} \frac{z^* s_i^{\text{out}} s_j^{\text{in}}}{1 + z^* s_i^{\text{out}} s_j^{\text{in}}} = L(G^*). \tag{19}$$

We can regard the fitness ansatz as restricting the maximum-likelihood principle to the parameter z only, since the original $2N$ Lagrange multipliers are assumed to be identifiable with (or approximated by) the empirical node strengths, up to a common parameter z. Indeed, applying the maximum-likelihood principle to z leads precisely to Eq. (19)[5].

Checking how good the fitness ansatz is for a given network (for which we know both the degrees and the strengths) can be done in two equivalent ways. Directly, we may explicitly find the values $\vec{\alpha}^*, \vec{\beta}^*$ solving the $2N$ coupled equations (16) and (17) and, then, check how good the relationships $e^{-\alpha_i^*} \propto s_i^{\text{out}}$ and $e^{-\beta_j^*} \propto s_j^{\text{in}}$ are. Alternatively, we may insert the ansatz $e^{-\alpha_i^*} \approx \sqrt{z^*} s_i^{\text{out}}$ and $e^{-\beta_j^*} \approx \sqrt{z^*} s_j^{\text{in}}$ into Eqs. (16) and (17) and check how well the empirical degrees are approximated by the expected degrees under the dcGM, i.e. how well the following relationships hold:

$$\langle k_i^{in} \rangle^{\text{dcGM}} = \sum_{j(\neq i)} \frac{z^* s_j^{\text{out}} s_i^{\text{in}}}{1 + z^* s_j^{\text{out}} s_i^{\text{in}}} \approx k_i^{in}(G^*), \quad \forall i, \tag{20}$$

$$\langle k_i^{out} \rangle^{\text{dcGM}} = \sum_{j(\neq i)} \frac{z^* s_i^{\text{out}} s_j^{\text{in}}}{1 + z^* s_i^{\text{out}} s_j^{\text{in}}} \approx k_i^{out}(G^*), \quad \forall i. \tag{21}$$

Notice that the latter procedure does not require solving Eqs. (16) and (17) but only the single equation (19) setting z^*. Since we are dealing with a network where N is very large, in our analysis we adopt the latter test.

It is straightforward to replicate the steps leading to Eq. (12) in the case of the DCM, irrespectively of the values of $\vec{\alpha}$ and $\vec{\beta}$:

$$S(\vec{\alpha}, \vec{\beta}) = \sum_{i=1}^{N} (\alpha_i \langle k_i^{out} \rangle + \beta_i \langle k_i^{in} \rangle) + \ln Z(\vec{\alpha}, \vec{\beta}); \tag{22}$$

now, if we insert the values $\vec{\alpha}^*, \vec{\beta}^*$ solving Eqs. (16) and (17), we get the exact relationship between entropy and minus log-likelihood as in Eq. (13):

$$S(\vec{\alpha}^*, \vec{\beta}^*) = \sum_{i=1}^{N} [\alpha_i^* k_i^{out}(G^*) + \beta_i^* k_i^{in}(G^*)] + \ln Z(\vec{\alpha}^*, \vec{\beta}^*) = -\mathscr{L}(G^*|\vec{\alpha}^*, \vec{\beta}^*); \tag{23}$$

if, instead, we plug the values $\tilde{\alpha}_i = -\ln(\sqrt{z^*} s_i^{out})$ and $\tilde{\beta}_i = -\ln(\sqrt{z^*} s_i^{in})$ implied by the fitness ansatz, we obtain

$$S(\vec{\tilde{\alpha}}, \vec{\tilde{\beta}}) = \sum_{i=1}^{N} [\tilde{\alpha}_i \langle k_i^{out} \rangle^{\text{dcGM}} + \tilde{\beta}_i \langle k_i^{in} \rangle^{\text{dcGM}}] + \ln Z(\vec{\tilde{\alpha}}, \vec{\tilde{\beta}}) = -\mathscr{L}(G^*|\vec{\tilde{\alpha}}, \vec{\tilde{\beta}}), \tag{24}$$

where $\mathscr{L}(G^*|\vec{\tilde{\alpha}}, \vec{\tilde{\beta}})$ is exactly the log-likelihood of the dcGM model, while $S(\vec{\tilde{\alpha}}, \vec{\tilde{\beta}})$ is the entropy of a DCM with degrees $\langle \vec{k}^{out} \rangle^{\text{dcGM}}$ and $\langle \vec{k}^{in} \rangle^{\text{dcGM}}$, i.e. the maximum-entropy ensemble with degrees given by the fitness ansatz in Eqs. (20) and (21).

This relationship holds for the scGM as well; however, the fitness ansatz is no longer defined at the degree level but at the degree per sector level. A clearer way to imagine this is to consider the multi-layer description discussed in section S.1: there, the fitness ansatz applies to each layer independently. To simplify the notation, let Γ be the set of all possible industries (or products) and $\gamma \in \Gamma$ denote a specific layer with only edges that come from a node of that sector. We can, then, write equivalently $s_{g_i \to j}$ or $s_j^{\text{in},\gamma}$ given that $g_i = \gamma$. To avoid confusion here we note, as in section S.1, that we can write equivalently also s_i^{out} or $s_i^{\text{out},\gamma}$ if $g_i = \gamma$, given the way layers are constructed. Then, we can say that the expected degrees in the scGM are given by:

$$\langle k_i^{in} \rangle^{\text{scGM}} = \sum_{\gamma \in \Gamma} \langle k_i^{in,\gamma} \rangle^{\text{scGM}} = \sum_{\gamma \in \Gamma} \sum_{\{j: j \neq i, g_j = \gamma\}} \frac{z^* s_j^{\text{out},\gamma} s_i^{\text{in},\gamma}}{1 + z^* s_j^{\text{out},\gamma} s_i^{\text{in},\gamma}}, \quad \forall i, \tag{25}$$

$$\langle k_i^{out} \rangle^{\text{scGM}} = \sum_{\gamma \in \Gamma} \langle k_i^{out,\gamma} \rangle^{\text{scGM}} = \sum_{\gamma \in \Gamma} \sum_{\{j: j \neq i, g_j = \gamma\}} \frac{z^* s_i^{\text{out},\gamma} s_j^{\text{in},\gamma}}{1 + z^* s_i^{\text{out},\gamma} s_j^{\text{in},\gamma}}, \quad \forall i. \tag{26}$$

From the equations above we can clearly see that this is equivalent to applying the fitness ansatz on each layer independently and, as such, we must define the DCM on each layer as well. In this case, we can see that for each layer we will have two vectors of parameters $\vec{\alpha}_\gamma, \vec{\beta}_\gamma$ that in the DCM will be fitted in order to ensure that $\langle k_i^{out,\gamma} \rangle$ and $\langle k_i^{in,\gamma} \rangle$ are equal to the empirical values. For the scGM we can define a new set of values $\vec{\tilde{\alpha}}_\gamma, \vec{\tilde{\beta}}_\gamma$ given by $\tilde{\alpha}_{i,\gamma} = -\ln(\sqrt{z^*} s_i^{\text{out},\gamma})$ and $\tilde{\beta}_{i,\gamma} = -\ln(\sqrt{z^*} s_i^{\text{in},\gamma})$.

Upon substituting these $\vec{\tilde{\alpha}}_\gamma, \vec{\tilde{\beta}}_\gamma$ values into (22), we obtain the entropy of each layer. By noticing that the layers do not interact, then the total entropy is just the sum of each layer's entropy, which is given by:

$$\sum_{\gamma \in \Gamma} S(\vec{\tilde{\alpha}}_\gamma, \vec{\tilde{\beta}}_\gamma) = \sum_{\gamma \in \Gamma} \sum_{i=1}^{N} [\tilde{\alpha}_{i,\gamma} \langle k_i^{out,\gamma} \rangle^{\text{scGM}} + \tilde{\beta}_{i,\gamma} \langle k_i^{in,\gamma} \rangle^{\text{scGM}}] + \ln Z(\vec{\tilde{\alpha}}, \vec{\tilde{\beta}}) = -\mathscr{L}(G^*|\vec{\tilde{\alpha}}; \vec{\tilde{\beta}}), \tag{27}$$

just like in the case of the dcGM, $\mathscr{L}(G^*|\vec{\tilde{\alpha}}, \vec{\tilde{\beta}})$ is the log-likelihood of the fitted scGM model and $S(\vec{\tilde{\alpha}}_\gamma, \vec{\tilde{\beta}}_\gamma)$ is the entropy of the DCM model applied to layer γ with expected degrees $\langle k_i^{in,\gamma} \rangle^{\text{scGM}}$ and $\langle k_i^{out,\gamma} \rangle^{\text{scGM}}$.

It is, therefore, true that in both models the negative log-likelihood $-\mathscr{L}(G^*|\vec{\tilde{\alpha}}, \vec{\tilde{\beta}})$ is still a measure of the number of alternative network configurations having those degrees as expected values. If Eqs. (20) and (21), or (25) and (26), are well obeyed by the data, then $-\mathscr{L}(G^*|\vec{\tilde{\alpha}}, \vec{\tilde{\beta}})$ is also a good approximation of the original entropy $S(\vec{\alpha}^*, \vec{\beta}^*)$, i.e. of the number of network configurations having the empirical degrees as expected values.

S.3 Relationship with block models

Let us, now, explore the relationship between the scGM and block models, specifically the Block Configuration Model (BCM)[6]. To derive the BCM, let us start from the definition of the contribution of each group to the nodes degrees. We can express these contributions as

$$k_i^{r\to s} = \delta_{g_i r} \sum_{j(\neq i)} \delta_{g_j s} a_{ij}, \tag{28}$$

$$h_i^{r\to s} = \delta_{g_i r} \sum_{j(\neq i)} \delta_{g_j s} a_{ji}; \tag{29}$$

in line with the discussion in section S.2 it is possible to define an Hamiltonian accounting for the quantities above and that results in the probability of a link existing from node i in group g_i to node j in group g_j reading

$$p_{ij} = \frac{e^{-\alpha_i^{g_i\to g_j} - \beta_j^{g_i\to g_j}}}{1+e^{-\alpha_i^{g_i\to g_j} - \beta_j^{g_i\to g_j}}}. \tag{30}$$

The multipliers $\alpha_i^{g_i\to g_j}$ and $\beta_j^{g_i\to g_j}$ can, then, be found by imposing that the expected value of the constrained quantities equals their empirical value, that is:

$$\langle k_i^{r\to s}\rangle = \delta_{g_i r} \sum_{j(\neq i)} \delta_{g_j s} p_{ij} = k_i^{r\to s}, \tag{31}$$

$$\langle h_i^{r\to s}\rangle = \delta_{g_i r} \sum_{j(\neq i)} \delta_{g_j s} p_{ji} = h_i^{r\to s}. \tag{32}$$

We can clearly see that if we constrain the values of the multipliers of the BCM with the following fitness ansatz

$$\alpha_i^{g_i\to g_j} = -\ln\left(\sqrt{z} s_i^{\text{out}}\right), \tag{33}$$

$$\beta_j^{g_i\to g_j} = -\ln\left(\sqrt{z} s_{g_i\to j}\right) \tag{34}$$

we recover the p_{ij} equation of the scGM. It is, therefore, possible to argue that the scGM is derived from the BCM in the same sense it is derived from the DCM: however, this is true only for the specific case of single product firms - that should be taken as a consequence of the available data rather than the approach. Our methodology, as discussed in section S.1, can be easily generalized to the multi-product case that requires us to consider a multi-layer network. We therefore consider the scGM a fitness-induced multi-layer DCM rather than a special case of the BCM.

Another approach present in the literature that has a strong resemblance to ours is the one using of a fitness model (dcGM) with a varying density that depends on the groups[7]. It modifies the dcGM link probability equation as follows

$$p_{ij} = \frac{z_{g_i g_j} s_i^{\text{out}} s_j^{\text{in}}}{1+z_{g_i g_j} s_i^{\text{out}} s_j^{\text{in}}} \tag{35}$$

where the $z_{g_i g_j}$ parameter is tuned to the desired density of connections between the two groups g_i and g_j. In our context, this would be achieved by setting $z_{g_i g_j}$ to satisfy

$$\langle L_{g_r g_s}\rangle = \sum_i \sum_{j(\neq i)} \delta_{g_i r}\delta_{g_j s} p_{ij} = \sum_i \sum_{j(\neq i)} \delta_{g_i r}\delta_{g_j s} a_{ij} = L^*_{g_r g_s} \quad \forall g_r, g_s \tag{36}$$

This is different from the scGM in three important ways. The first one concerns the link densities to be preserved: the scGM only considers L_{g_i} and not $L_{g_i g_j}$; this implies that while the block-wise dcGM ensures that the probability of connection depends on the number of links between the two groups, the scGM considers only the density of outgoing links from g_i independently of the group the edges are going to. This difference is however not at the core of the scGM methodology as it could be removed by letting the z depend on both groups also in the scGM.

A second minor difference concerns weights: the block-wise dcGM weights are assigned with the deterministic recipe $w_{ij} = \frac{s_i^{\text{out}} s_j^{\text{in}}}{w^{\text{tot}} p_{ij}} a_{ij}$. This creates a strange paradox: given a node in group g_i and two nodes in groups g_j and g_k with equal

in-strength $s_j^{\text{in}} = s_k^{\text{in}}$, the weight of the sampled edge will be higher for the node pair with lower probability of connection. This is not a desired property as it runs contrary to the fitness ansatz at the core of the methodology. This issue is resolved in the scGM by letting the weight depend on the strengths by sectors rather than the total ones. This is informative of the more fundamental distinction between the two approaches: the block-wise dcGM does not consider the heterogeneity that might exist between firms in the same group. Indeed by having p_{ij} depending only on $z_{g_i g_j}$ and the total strengths, there is no information on the production technology of each individual firm in terms of the product composition of its inputs. As such, in the block-wise dcGM, a link between firm i and j can exist even if firm j does not have any input from sector g_i so long as there exists a firm in g_j that does. As such, we think the block-wise dcGM to be unsuitable to analyse firm networks as it lacks some of the fundamental information necessary to correctly reproduce the true structure of production.

S.4 Fitness ansatz

Both the dcGM and the scGM rely on the simple hypothesis that strengths and degrees are positively correlated: if this were not the case for our datasets, both models would perform poorly. We qualitatively assess the veracity of this hypothesis by checking how well this correlation holds, at the various levels of sector definitions, in the empirical data. In figure S.1 (and figure S.8) we

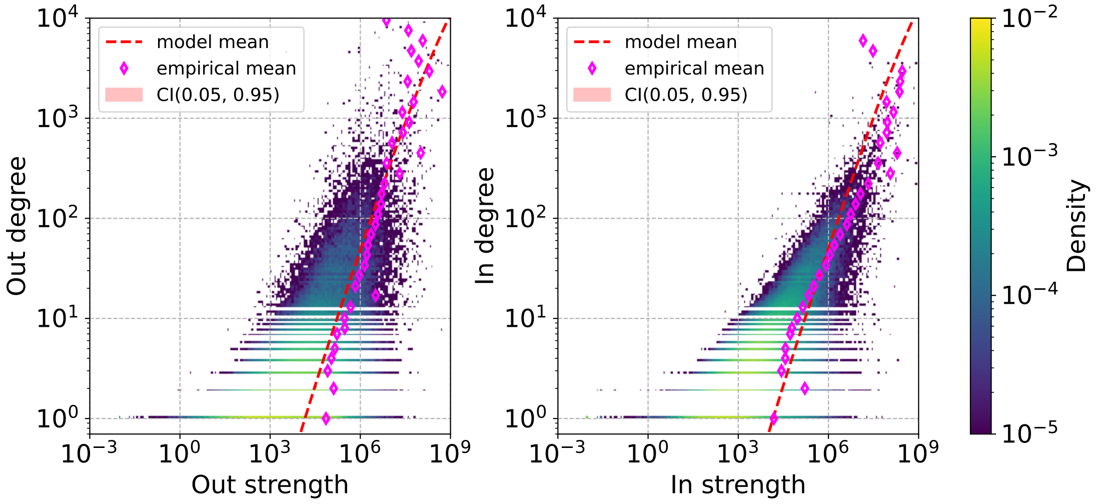

(a) All sectors

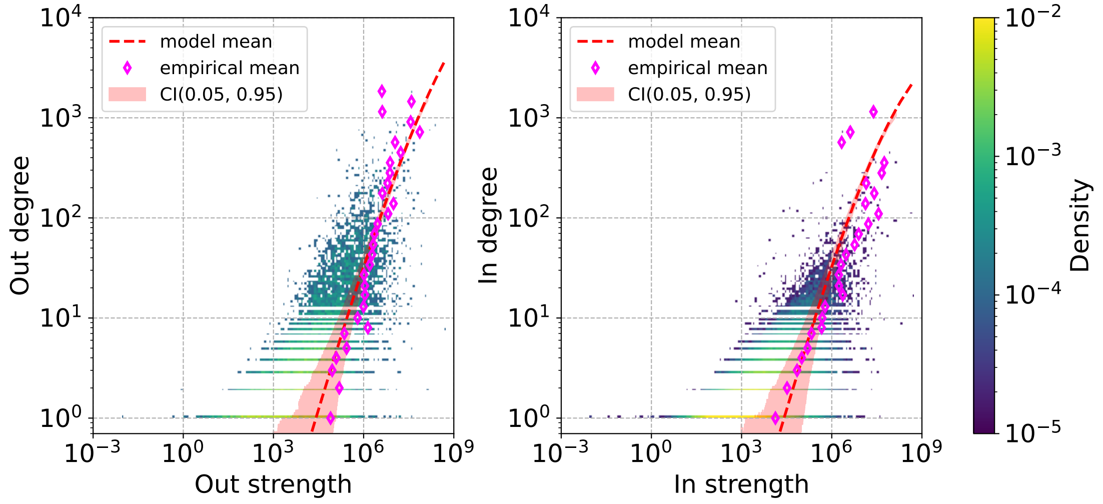

(b) C - Manufacturing

Figure S.1. (Continues)

plotted the relationship between out-strengths and out-degrees as well as between in-strengths and in-degrees by sector, for an increasingly specific sector definition. We find that the fitness ansatz is well supported by the data in terms of trend and average, with both models being able to correctly capture the trend at both the aggregate and sector-specific level. Notice, however, that the spread of data points around the mean is not reproduced by either model - the predicted variance of the degree being less than, or of the same order of magnitude of, its expected value; this discrepancy is, again, due to the fitness ansatz which leads our models to predict degrees that are, by construction, proportional to the strengths, hence the expected monotonic trend.

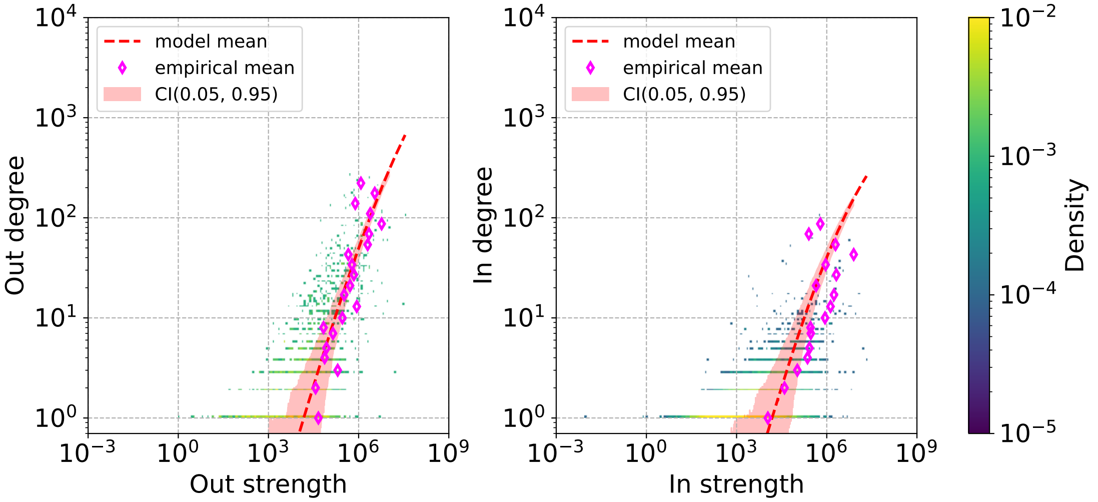

(c) 33 - Repair and installation of machinery and equipment

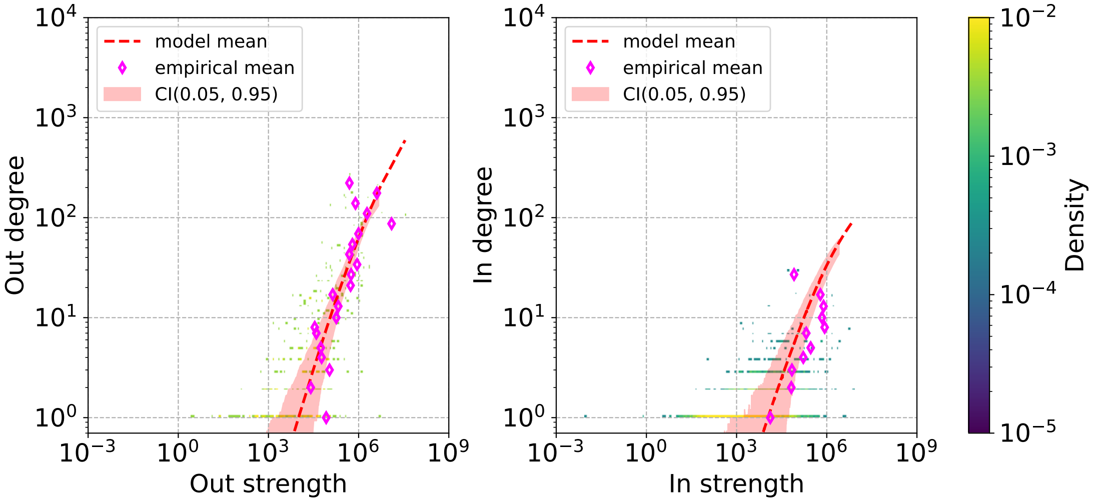

(d) 33121 - Repair and maintenance of machinery for general use and machine parts

Figure S.1. Degree as a function of strength at various hierarchical levels of a sector definition for the network of Institution 1. In each panel we are showing the degree/strength relation for the product layer specified. Note that edges exist on a layer if the node they come from belongs to the given sector. Coloured area represents the density of points in the empirical network for each bin of a two dimensional logarithmic binning. The purple rhombuses give the average empirical strengths computed over a logarithmic binning of the degree. The red dashed line shows the theoretical relationship between expected strength and degree in the fitted scGM. The red shaded area is computed by taking one hundred samples from the scGM ensemble and computing the 5th and 95th percentile value of the degree for each node and then smoothing the resulting points using a moving average over increasing values of strength. The area therefore represents the interval where 90% of sampled degree values will lie for a node of given strength.

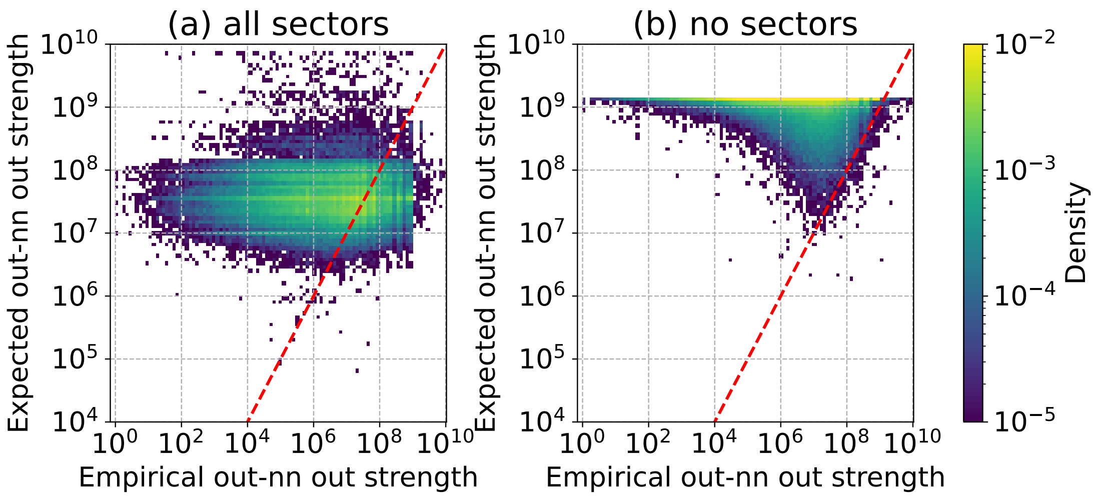

(a) Out-out

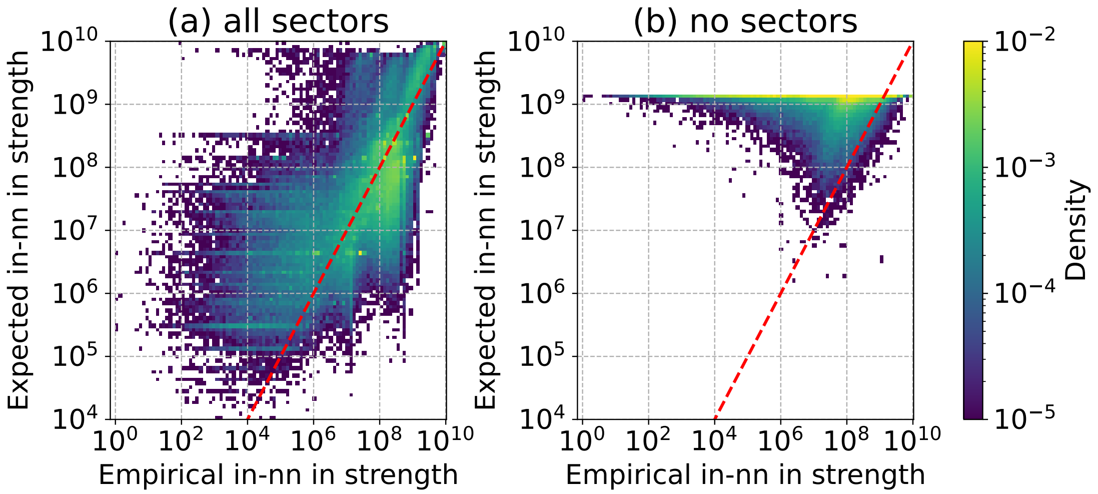

(b) In-in

Figure S.2. Per node comparison of the average nearest out-neighbour out-strength (a) and average nearest in-neighbour in-strength (b) for Institution 1. The shaded area represents the density of points in the two-dimensional logarithmic binning.

S.5 Structural properties

In the main text we discussed how the dcGM is not able to accurately reproduce the trend of the average nearest neighbour strength. We can observe this phenomenon also via a direct comparison between the expected average nearest neighbours strengths and the empirical ones. If the ensembles had captured the correct average size of companies in the neighbourhood of each node, then all points in figure S.2 would cluster around the red dashed line. However, both the right plots in figure S.2 show that the majority of nodes are assigned a high value of the average nearest neighbours strength because all nodes are more likely to connected to the nodes with the highest total strength. By introducing restrictions by sector, the scGM still preserves the same trend, which is intrinsic to the fitness ansatz, but only within the correct sector. This allows the scGM to predict the neighbours of the nodes and, hence, their properties more accurately. This difference between the dcGM and the scGM is even more evident in figure S.3 where we have repeated the same comparison per node but splitting each node in-strength by its sector components. This allows us to visualize the error in reconstructing the in-strength by sector of the in-neighbours of each node. It is evident that the scGM performs significantly better than the dcGM as the point are more closely packed around the 45 degree line.

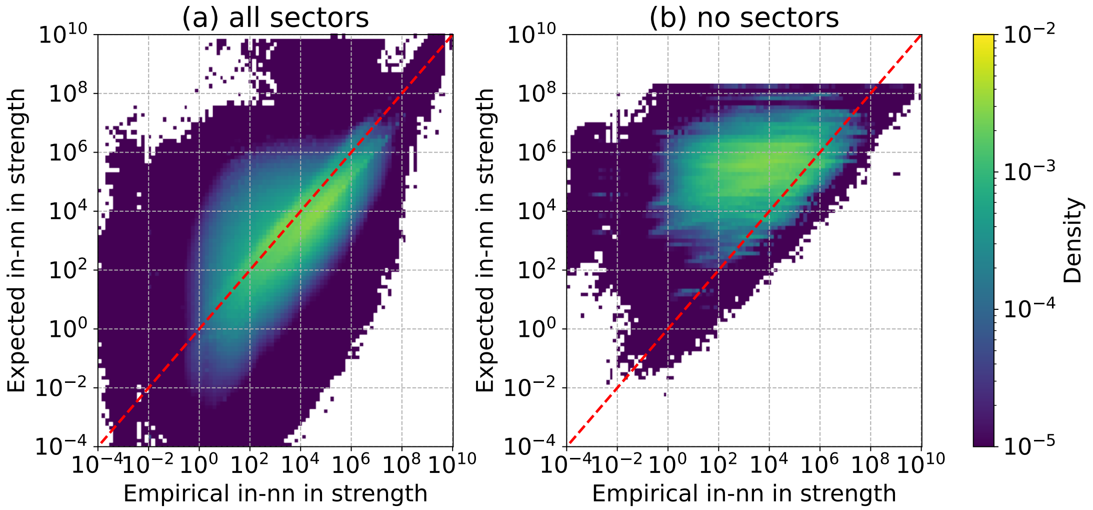

Figure S.3. Per node comparison of the average nearest in-neighbour in-strength by sector for Institution 1. Each firm has been divided into multiple points, one for each sector component. The shaded area represents the density of points in the two-dimensional logarithmic binning.

S.6 Identifying firms

The available data consists of all incoming and outgoing transactions on the accounts of the Dutch clients of the banks. As we are concerned only with inter-firm transactions we exclude from the analysis any account belonging to a private individual. For the ING data, it is known from ING accounts whether they belong to private individuals or clients. For the ABN AMRO dataset this is achieved using the Business Contact Database (BCDB) curated by the Commercial Banking (CB) department. In this database for each firm there is a hierarchical definition of the commercial complex it belongs to. This allows us to both remove private individuals that do not have an assigned commercial complex and to reconstruct the often complex hierarchical relationships that exists between accounts of the same firm. Note that there is a difference between the two approaches: the ING data is more inclusive guaranteeing a maximum coverage of all commercial clients; the ABN approach on the other hand is quite restrictive, any node included will definitely be a firm, however smaller firms might not be included if they have not been correctly labelled as complexes. The commercial complex is a definition that comes from the account manager for the commercial client that together with the client determines which accounts belong to the same firm and which among the available contacts is the main account holder. A similar structured grouping of accounts is achieved in the ING data using the legal (ultimate) parent information available in the transaction data from ING.

Each commercial complex will be a single node in our network and all transactions within the group will be discarded while transactions between groups will be summed irrespective of the specific account on which the transfer was recorded. This step of identifying firm is a delicate but fundamental step. The transaction data is per se already a network as it encodes flows of money from one account to another. However this data cannot be used as is, because it would not be an accurate description of the input-output relationships between firms. This is because we are interested in constructing an input-output network where each node is a functional economic unit such that any incoming money into the account can be interpreted as the remuneration for a product or service of a known type. In order to achieve this, two things must be true: first only transactions that correspond to final consumption or intermediate use of products should be included, and second the accounts must be aggregated such that all expenses and income relating to production are assigned to the same node. You can better understand the importance of the second point by imagining a firm with multiple accounts, one used to pay employees, taxes and receive sales income, and one to pay suppliers. If we considered these accounts as independent nodes we would have broken the functional economic unit and its single balance sheet into two meaningless units and would be considering transfers between accounts as a sale of some product. We could, of course, exaggerate in the other direction, that is by aggregating more than necessary. Consider the case of a financial holding that owns multiple businesses: each business has its own accounts and balance sheet and should be treated as an independent node, if we were to consider all firms owned by the holding as a single node we would be ignoring the fact that these balance sheets operate independently and their production processes are not the same. The commercial complex definition used in our analysis has been developed with a similar objective in mind: that is to be able to analyse the total operating income and expenses of a firm as a whole.

A final step in the identification of the firm is constituted by the correct assignment of an industry label. For the purpose of this analysis we use the Dutch Standaard Bedrijfsindeling (SBI) hierarchical five digit codes for the ABN AMRO data and the North American Industry Classification System (NAICS) hierarchical six digit codes for the ING data. Note that the SBI codes are well aligned with the Statistical Classification of Economic Activities in the European Community (NACE) codes, as they share the same first four digits, and the International Standard Industrial Classification of All Economic Activities (ISIC), as the first two digits are the same. All Dutch firms are assigned an SBI code when they register at the Chamber of Commerce (Kamer van Koophandel, KvK) however given the complex legal structure that companies may adopt, not always is it clear which of the different codes associated with a commercial complex is the main one. In order to address this internally at ABN AMRO to each commercial complex has been associated a 'real' SBI which the account manager believes best captures the real economic activity of the firm. This curated information is particularly useful as often firms have a legal structure with a financial holding at its apex, meaning we would see an over-representation of the financial sector. The reason for using the NAICS codes for ING is that a similar manual overwrite of the sector code is done at ING but only on the NAICS codes. We have chosen to give precedence to this better quality data rather than coherence between the two datasets as the analysis does not rest on the codes being the same for both networks and that they are quite similar classifications.

S.7 Filtering transactions

The bulk of transactions available in the two datasets is composed of SEPA payments. Both however also contain Swift, as well as other international and internal transfers related among others to repayment of loans, credit due to financial investments or deposit of a loan, cash and card payments. As discussed above we want to keep only the payments relating to the sales of the companies, as such we need to eliminate all transactions related to investments and credit, as well as all internal transfers between accounts. We do so by considering all transactions between firms to be related to an exchange of a product or service and by eliminating all firms from the financial and insurance sector (SBI codes starting with 64, 65 or 66, and NAICS codes starting with 52) and the public administration (SBI codes starting with 84, and NAICS codes starting with 92). The remaining transactions will be related to either final consumption or to other credit and debit relationships. For the purpose of this analysis it is however sufficient for us to use only the firm to firm connections and ignore the household consumption.

Another important aspect of the data is the temporal dimension. The available data is extremely granular, however for the purpose of understanding the structure of the relationship between firms we would like to have a more static view. As such we chose to aggregate transactions on a yearly basis, so that a link from node A to node B represents the total flow of goods produced by firm A and bought by firm B in a given year measured in Euros. A year is chosen as it will reduce any effect that seasonality may have giving us a balanced representation of all firms. Indeed, if we chose a monthly or quarterly aggregation those sectors that see most of their activity in a given period of the year would be underrepresented in certain snapshots.

S.8 Incomplete information

It should be noted that unfortunately we cannot claim to have complete coverage of all inter-client transactions. There are two main limitations to our data: mediated transfers and unobserved direct flows. The first case is possibly the more problematic as there exist many payment service providers as well as other systems that mediate between firms. In particular there exist various services offered by financial institutions to pay upfront all credit invoices, or parts of them, taking upon themselves the risk of the customer not paying or paying late. These kind of transactions are precisely what we would like to include in our analysis. We know that these transfers exist in our data and we can in some cases identify one side of the transaction on the accounts, however linking sender and receiver is not easy. Further work could be dedicated to understanding better this issue and how to solve it possibly developing an algorithm to find matching pairs of unconnected flows. The second issue relates to the heterogeneity in the payment systems available to the clients. Other than SEPA transfers we have batch payments, cash deposits and withdrawals, card payments and credit, international transfers, and many others. In the case of cash it is of course impossible to determine the origin of the payment, however also in many other cases in our datasets it is not possible to clearly identify the counterpart to a transaction.

Furthermore the effect of limiting ourselves to inter-client transactions will introduce a bias in our analysis that will vary firm by firm. The proportion of observed to unobserved links will be determined by how connected the client is to firms that are not part of the bank's network and to businesses abroad. Unfortunately we are currently not able to determine if those accounts belong to a private individual or a firm, nor group the accounts as discussed in section S.6. We are also not able to correctly assign an industrial classification code to those firms. How this will affect our analysis is not easy to determine. The quality of our reconstruction depends fundamentally on the functional relation there is between fitness and probability of a link existing. In our case, where the fitness is the strength of the node, we can see two possibilities for a given firm: we observe a high strength but a lower than expected degree because few counterparties to this firm are in the network, but the weight of the transaction that are observed represent the majority of the "true" strength of the firm; we observe a low strength

because the only transactions that are in our network are of low value, and hence the strength is not representative of the real probability of observing a link. Both cases are possible because we expect the weights of each link to be proportional to the size of both companies involved. Given the power law nature of the size of firms, we expect many transactions of low value and few that represent the majority of the strength. The second case in particular could result in fully disconnected nodes in our reconstructed samples and very low expected degree.

S.9 Descriptive statistics

The network constructed with the methodology so far described is composed of one giant connected component containing 99.56 % of nodes for the ABN data and 99.998% for the ING data. Removing the financial and government sectors does not affect significantly the number of components with the largest component still retaining 99.54% of all nodes for the ABN data and 99.01% for the ING data. In both cases the other nodes are mostly in dyadic relations with the maximum component size of 27 nodes. In the table below we have summarized the sector compositions of the two datasets. Note that some of the discrepancies are due to the difficulty in precisely translating between the two classifications standards.

Table S.1. Percentages of number of firms per highest level of industrial classification. We use here the definitions of the NACE system and adapt the NAICS to these areas.

Symbol	Area	ABN	ING
A	AGRICULTURE, FORESTRY AND FISHING	2.83	1.10
B	MINING AND QUARRYING	0.10	0.07
C	MANUFACTURING	4.28	4.59
D	ELECTRICITY, GAS, STEAM AND AIR CONDITIONING SUPPLY	0.10	0.19
E	WATER SUPPLY; SEWERAGE, WASTE MANAGEMENT AND REMEDIATION ACTIVITIES	0.16	0.02
F	CONSTRUCTION	10.40	4.53
G	WHOLESALE AND RETAIL TRADE; REPAIR OF MOTOR VEHICLES AND MOTORCYCLES	16.05	15.24
H	TRANSPORTATION AND STORAGE	3.16	2.94
I	ACCOMMODATION AND FOOD SERVICE ACTIVITIES	3.92	3.84
J	INFORMATION AND COMMUNICATION	5.14	1.50
K	FINANCIAL AND INSURANCE ACTIVITIES	5.29	7.99
L	REAL ESTATE ACTIVITIES	2.40	11.49
M	PROFESSIONAL, SCIENTIFIC AND TECHNICAL ACTIVITIES	16.49	12.71
N	ADMINISTRATIVE AND SUPPORT SERVICE ACTIVITIES	5.14	3.80
O	PUBLIC ADMINISTRATION AND DEFENCE; COMPULSORY SOCIAL SECURITY	0.00	0.00
P	EDUCATION	2.83	1.63
Q	HUMAN HEALTH AND SOCIAL WORK ACTIVITIES	4.67	5.29
R	ARTS, ENTERTAINMENT AND RECREATION	4.16	6.23
S	OTHER SERVICE ACTIVITIES	9.81	16.86
T	ACTIVITIES OF HOUSEHOLDS AS EMPLOYERS; UNDIFFERENTIATED GOODS- AND SERVICES-PRODUCING ACTIVITIES OF HOUSEHOLDS FOR OWN USE	0.01	n/a
U	ACTIVITIES OF EXTRATERRITORIAL ORGANISATIONS AND BODIES	0.04	n/a

References

1. Jaynes, E. T. Information theory and statistical mechanics. *Phys. review* **106**, 620 (1957).

2. Squartini, T., Caldarelli, G., Cimini, G., Gabrielli, A. & Garlaschelli, D. Reconstruction methods for networks: the case of economic and financial systems. *Phys. reports* **757**, 1–47 (2018).

3. Cimini, G., Squartini, T., Gabrielli, A. & Garlaschelli, D. Estimating topological properties of weighted networks from limited information. *Phys. Rev. E* **92**, 040802 (2015).

4. Squartini, T. & Garlaschelli, D. Analytical maximum-likelihood method to detect patterns in real networks. *New J. Phys.* **13**, 083001 (2011).

5. Garlaschelli, D. & Loffredo, M. I. Maximum likelihood: Extracting unbiased information from complex networks. *Phys. Rev. E* **78**, 015101 (2008).

6. van Lidth de Jeude, J., Di Clemente, R., Caldarelli, G., Saracco, F. & Squartini, T. Reconstructing mesoscale network structures. *Complexity* **2019** (2019).

7. Ramadiah, A. *et al.* Network sensitivity of systemic risk. *arXiv preprint arXiv:1805.04325* (2018).

S.10 Other measures and complementary plots for Institution 2

We include for completeness other measures computed on the networks and fitted models.

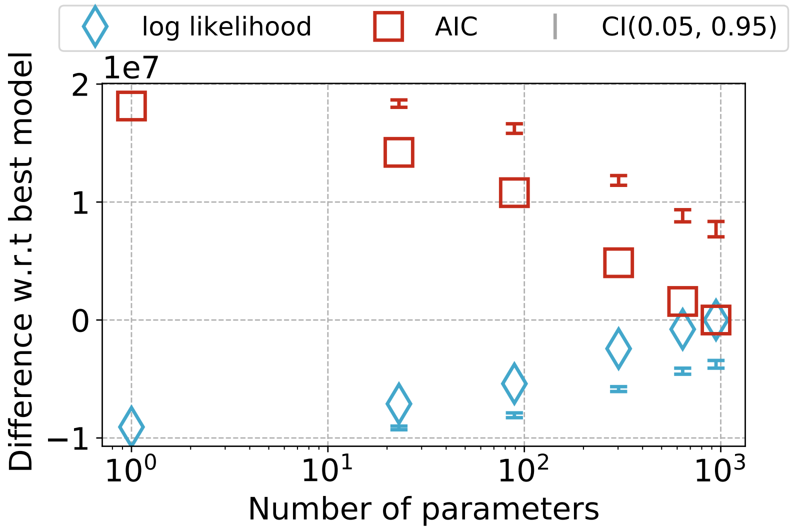

Figure S.4. Difference in log likelihood and AIC of the fitted models for an increasing number of sectors (layers) for the data of Institution 2. The results refer to the dcGM when only one layer exists and to the scGM otherwise. The results are given with respect to the best performing model. The error bars show the interval of log likelihoods of the fitted scGM models where the sector labels have been randomized.

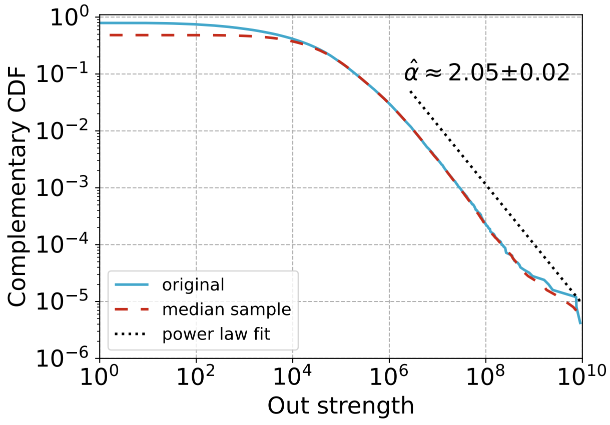

(a) Institution 1

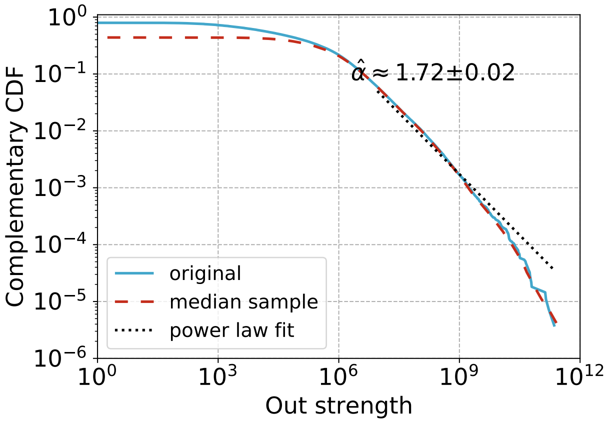

(b) Institution 2

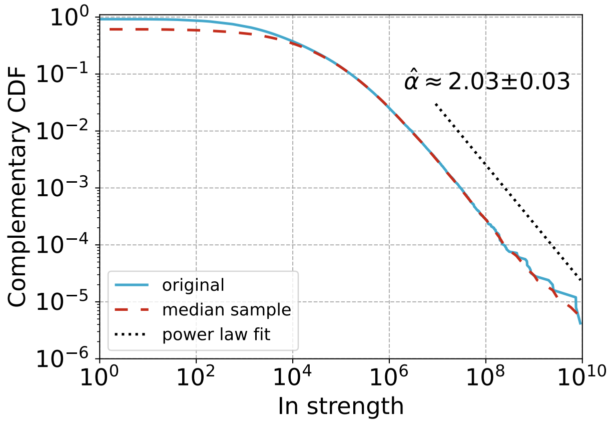

(c) Institution 1

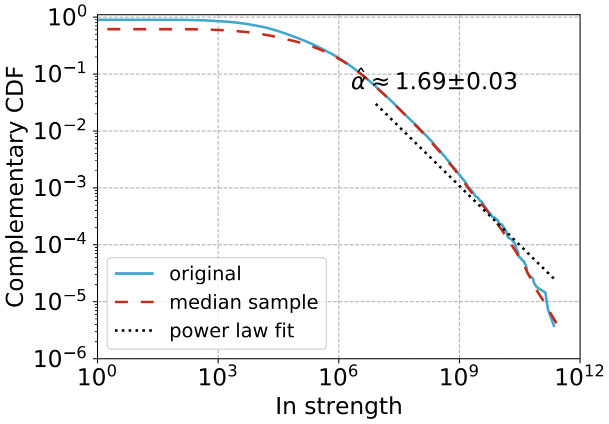

(d) Institution 2

Figure S.5. Complementary cumulative distribution of the out- and in-strength for institution 1 ((a) and (c)) and institution 2 ((b) and (d)). The median sample line is computed by taking 100 samples from the scGM ensemble with all sector and computing the median strength for each node and then the distribution of these median values. This allows us to see the distribution of the strength for a typical sample, highlighting for example the lower probability in the scGM of observing low strength nodes. We find that the distributions of institution 1 are almost as likely to follow a power law or a log-normal distribution but with slight preference for a log-normal (p-value 0.67 for (a)) and for power law (p-value 0.90 for (c)). For institution 2 we find a statistically significant support for log-normality (p-value $2.2 \cdot 10^{-5}$ for (b) and $2.4 \cdot 10^{-8}$ for (d)). Here, the null hypothesis is that the power-law performs better than a log-normal distribution.

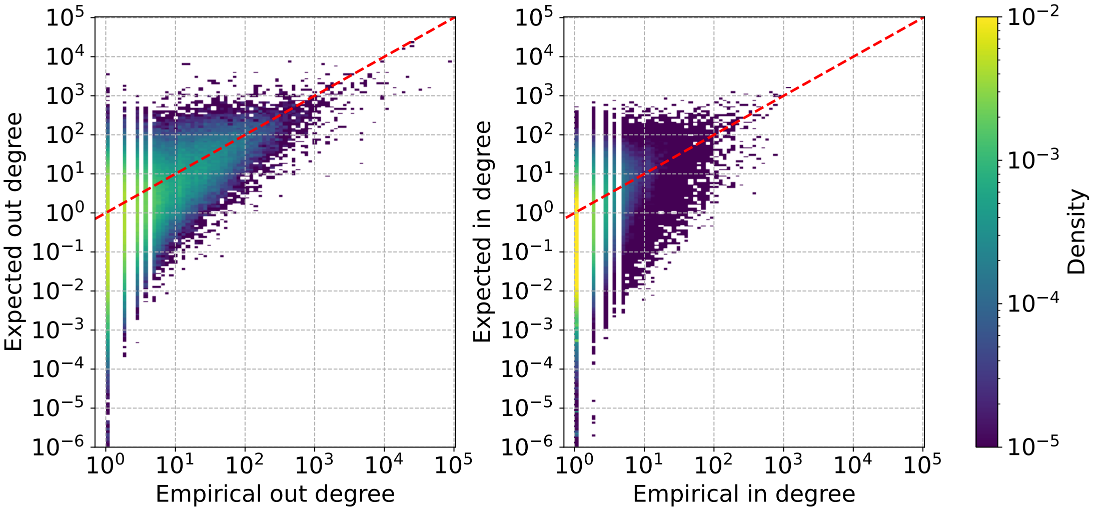

(a) scGM

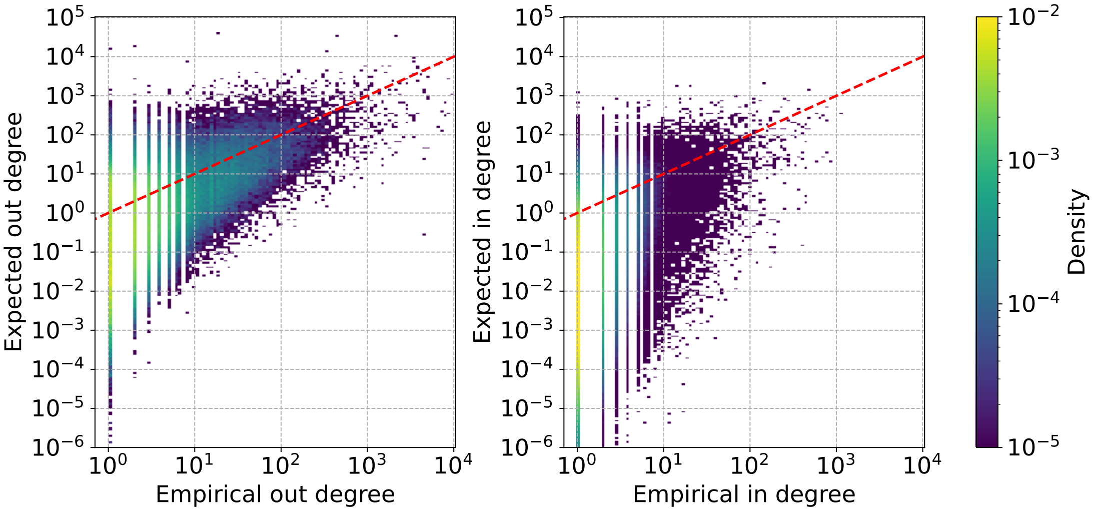

(b) dcGM

Figure S.6. Empirical versus expected degree by sector of the dcGM (a) and scGM (b) for the network of Institution 1.

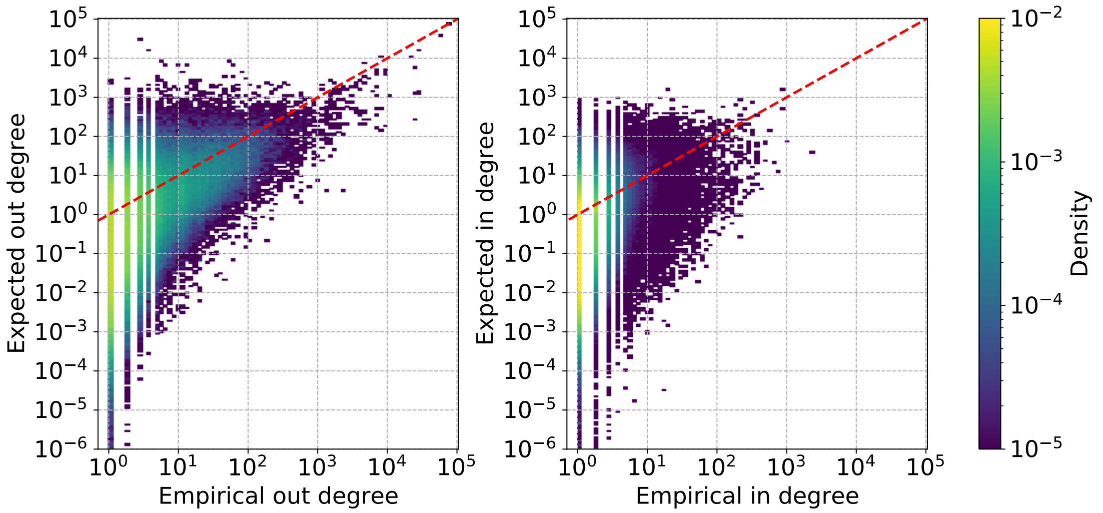

(a) scGM

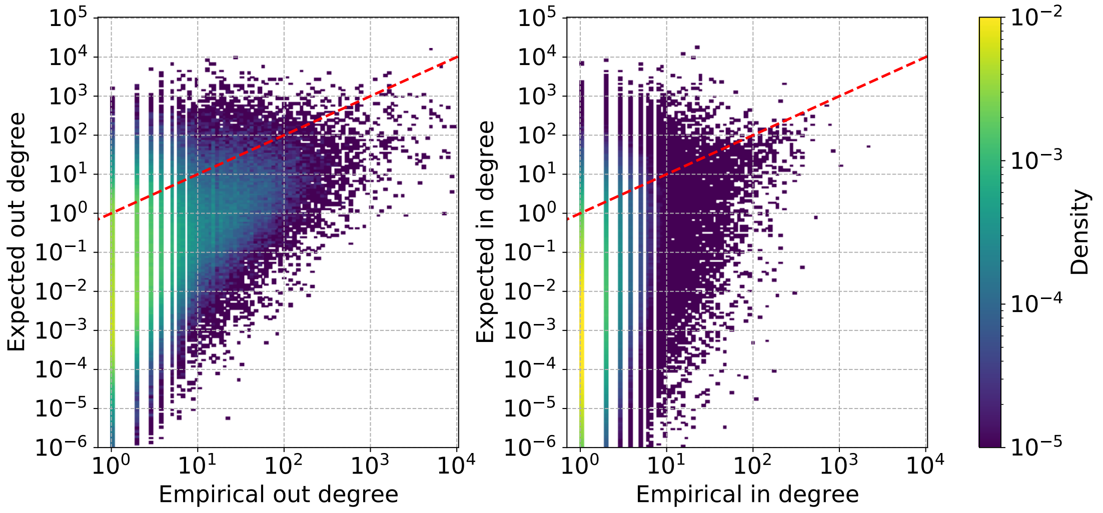

(b) dcGM

Figure S.7. Empirical versus expected degree by sector of the dcGM (a) and scGM (b) for the network of Institution 2.

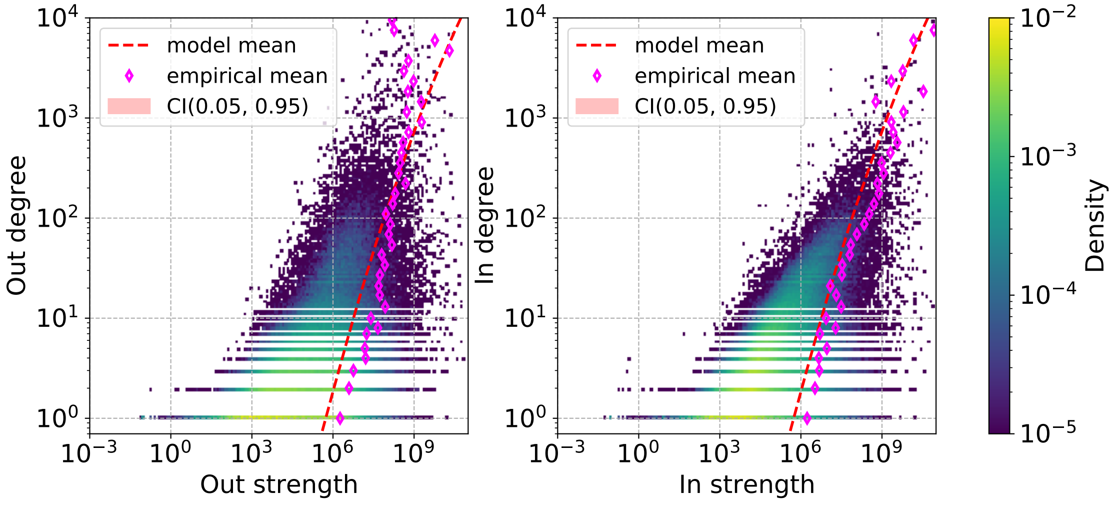

(a) All sectors

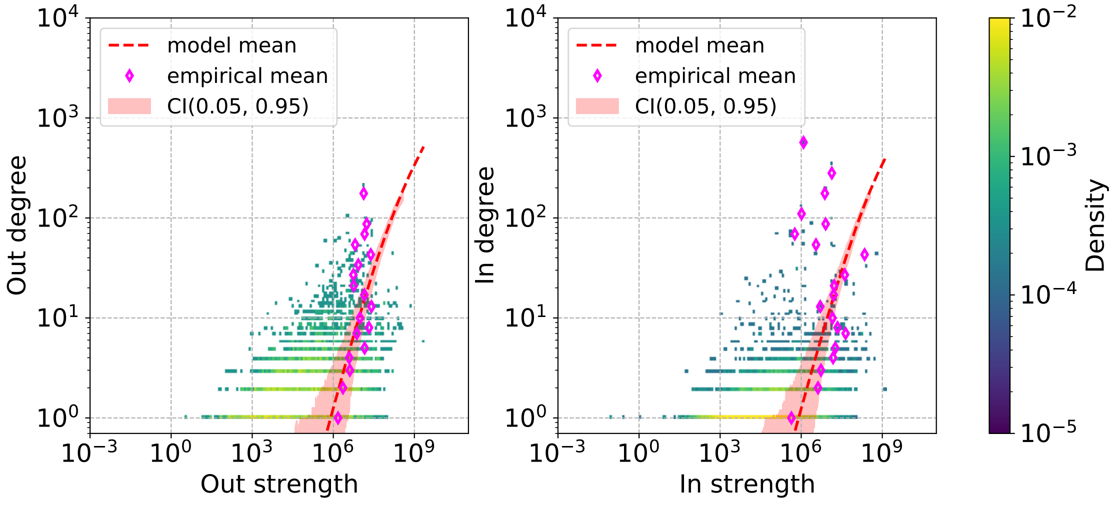

(b) C - Manufacturing

Figure S.8. (Continues)

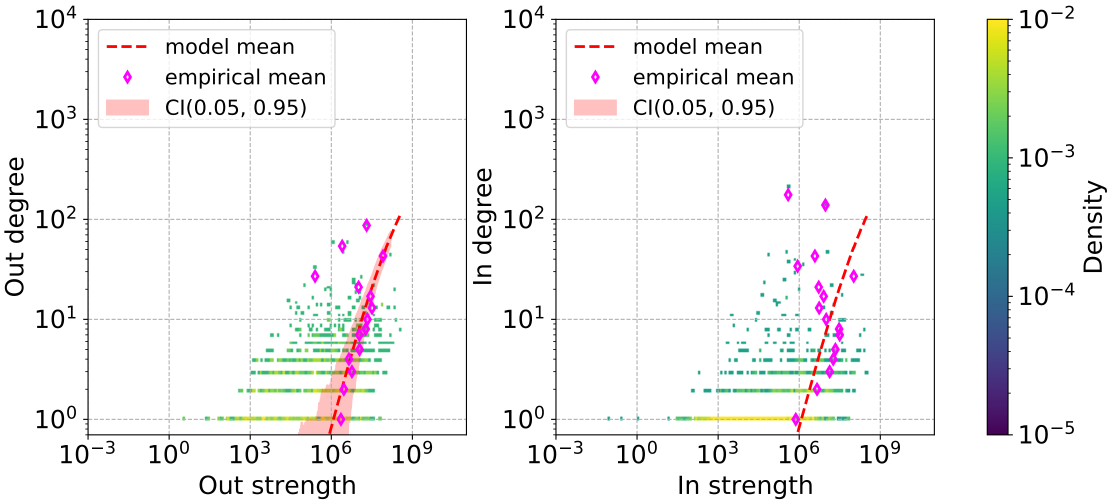

(c) 33 - Repair and installation of machinery and equipment

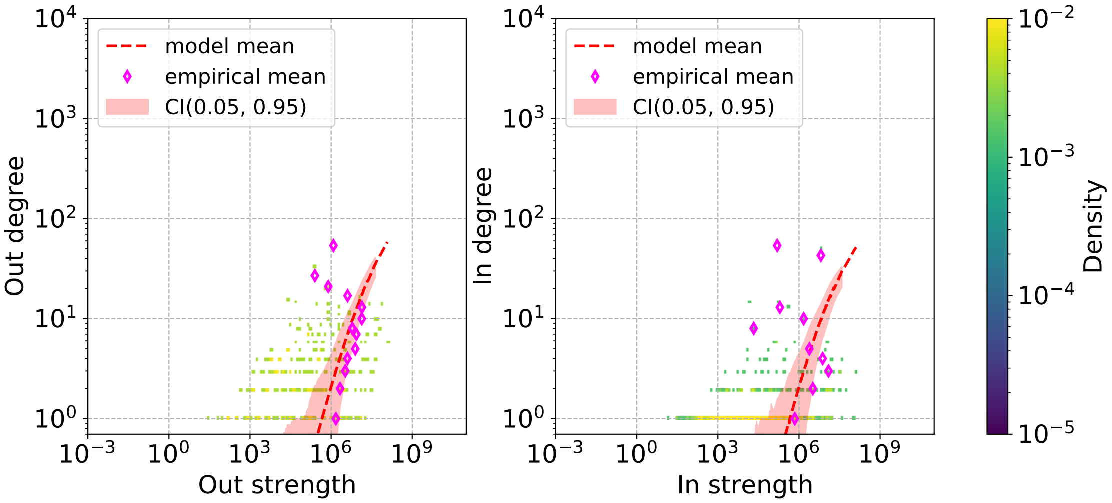

(d) 33121 - Repair and maintenance of machinery for general use and machine parts

Figure S.8. Degree as a function of strength at various hierarchical levels of a sector definition for the network of Institution 1. In each panel we are showing the degree/strength relation for the product layer specified. Note that edges exist on a layer if the node they come from belongs to the given sector. Coloured area represents the density of points in the empirical network for each bin of a two dimensional logarithmic binning. The purple rhombuses give the average empirical strengths computed over a logarithmic binning of the degree. The red dashed line shows the theoretical relationship between expected strength and degree in the fitted scGM. The red shaded area is computed by taking one hundred samples from the scGM ensemble and computing the 5th and 95th percentile value of the degree for each node and then smoothing the resulting points using a moving average over increasing values of strength. The area therefore represents the interval where 90% of sampled degree values will lie for a node of given strength.

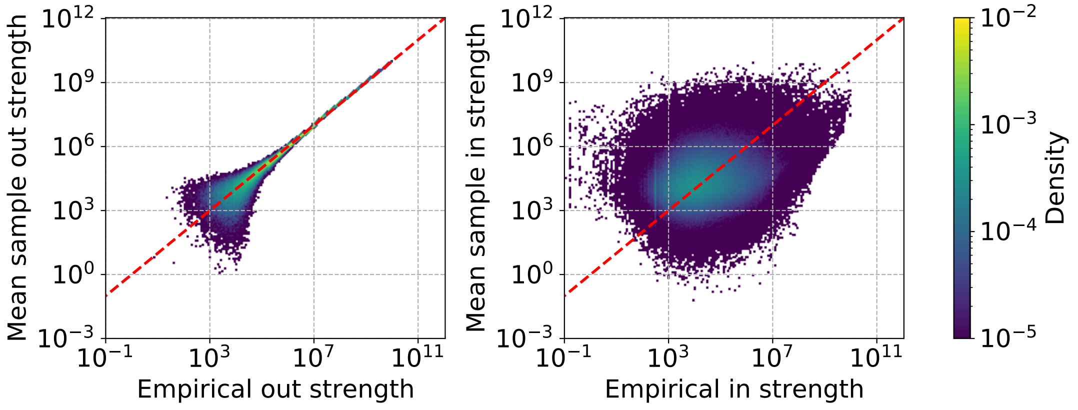

(a) dcGM

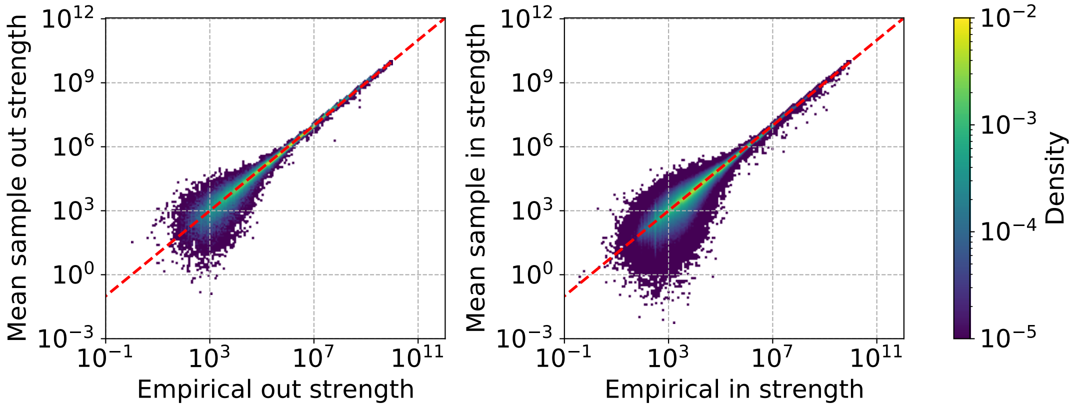

(b) scGM

Figure S.9. Empirical versus mean node strength by sector computed over 100 samples drawn from the dcGM (a) and scGM (b) ensembles for the network of Institution 2.

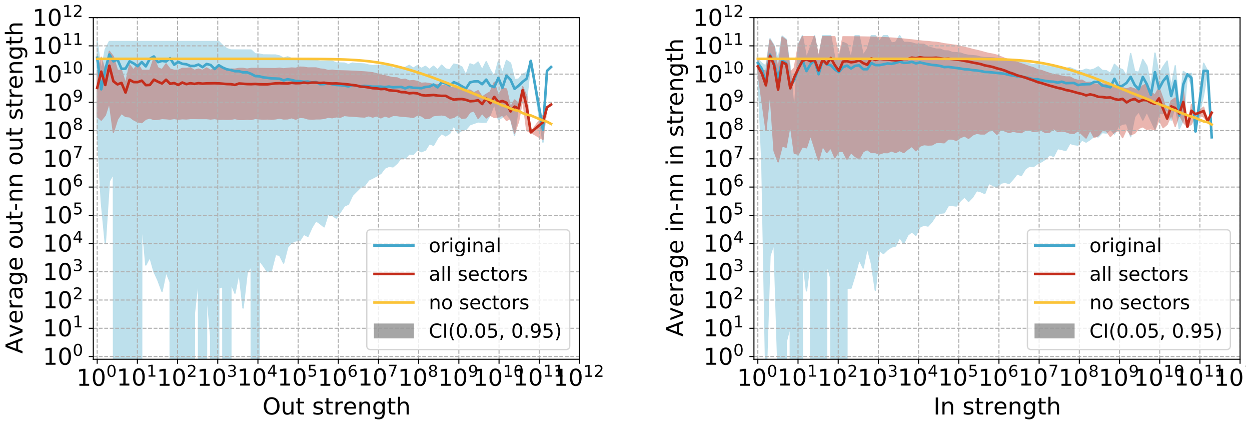

(a) Out-out

(b) In-in

Figure S.10. Average nearest out-neighbour out-strength by node out-strength (a) and average nearest in-neighbour in-strength by node in-strength (b) for Institution 2. The full lines are computed as the average over all firms in the logarithmic binning of the x-axis. The confidence interval is the empirical interval measured over the same bins.

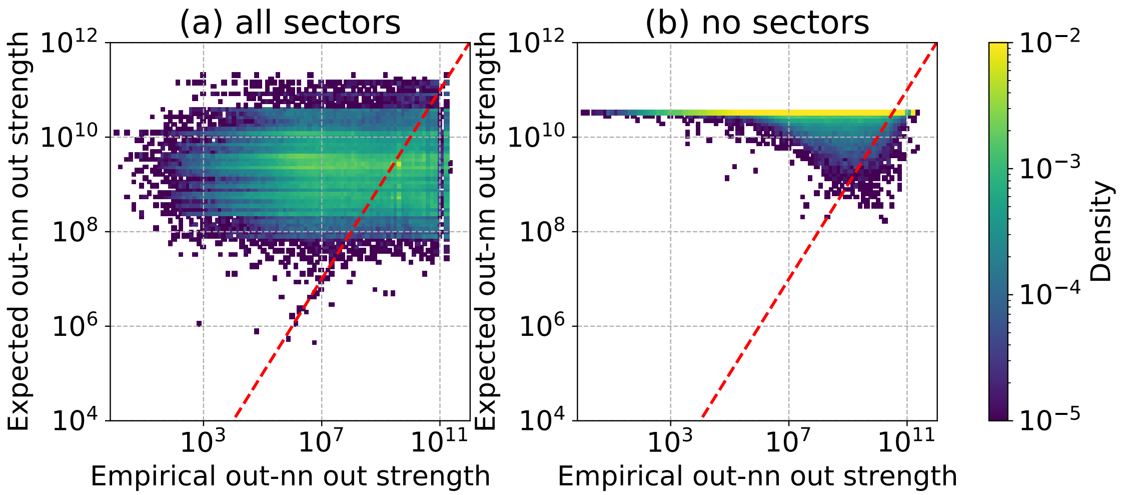

(a) Out-out

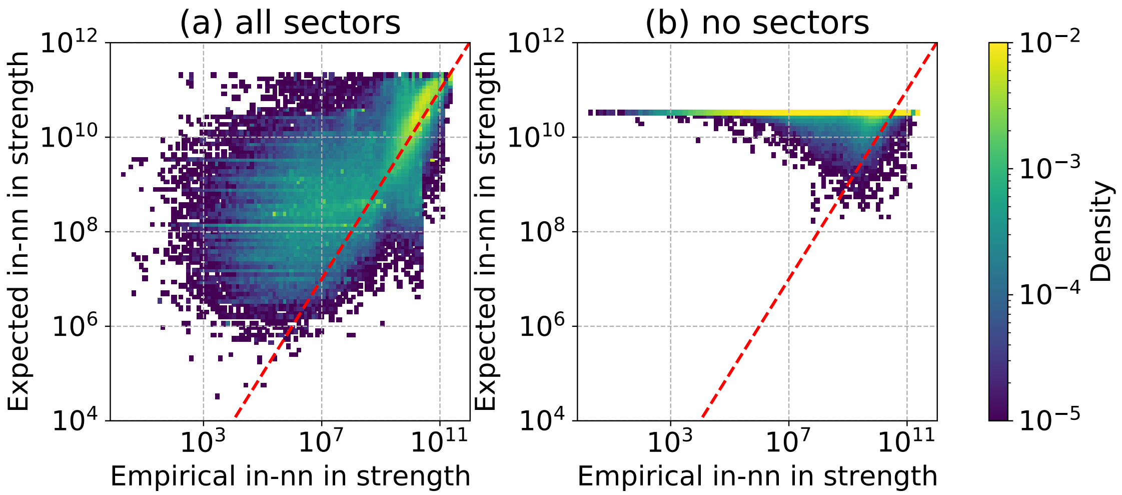

(b) In-in

Figure S.11. Per node comparison of the average nearest out-neighbour out-strength (a) and average nearest in-neighbour in-strength by node in-strength (b) for Institution 2. The shaded area represents the density of points in the two-dimensional logarithmic binning.

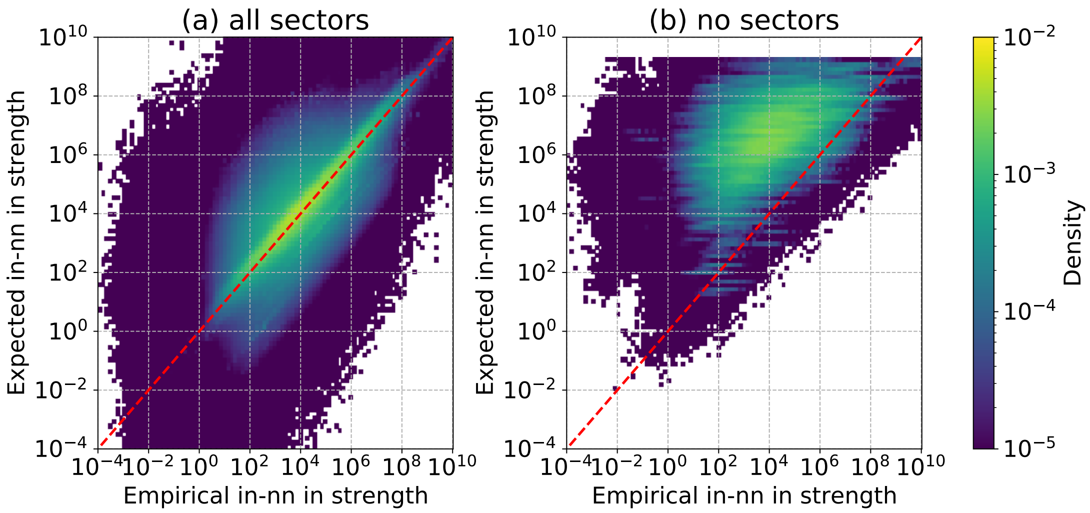

Figure S.12. Per node comparison of the average nearest in-neighbour in-strength by sector for Institution 2. Each firm has been divided into multiple points, one for each sector component. The shaded area represents the density of points in the two-dimensional logarithmic binning.
